# Prevalence of and reasons for women’s, family members’, and health professionals’ preferences for cesarean section in China: A mixed-methods systematic review

**DOI:** 10.1371/journal.pmed.1002672

**Published:** 2018-10-16

**Authors:** Qian Long, Carol Kingdon, Fan Yang, Michael Dominic Renecle, Shayesteh Jahanfar, Meghan A. Bohren, Ana Pilar Betran

**Affiliations:** 1 Global Health Research Center, Duke Kunshan University, Kunshan, Jiangsu Province, China; 2 School of Community Health and Midwifery, University of Central Lancashire, Preston, United Kingdom; 3 Johns Hopkins Bloomberg School of Public Health, Baltimore, Maryland, United States of America; 4 College of Osteopathic Medicine, Des Moines University, Des Moines, Iowa, United States of America; 5 School of Public Health, Central Michigan University, Mount Pleasant, Michigan, United States of America; 6 Gender and Women’s Health Unit, Centre for Health Equity, Melbourne School of Population and Global Health, University of Melbourne, Carlton, Victoria, Australia; 7 UNDP/UNFPA/UNICEF/WHO/World Bank Special Programme of Research, Development and Research Training in Human Reproduction (HRP), Department of Reproductive Health and Research, World Health Organization, Geneva, Switzerland; King's College London, UNITED KINGDOM

## Abstract

**Background:**

China has witnessed a rapid increase of cesarean section (CS) rates in recent years. Several non-clinical factors have been cited as contributing to this trend including maternal request and perceived convenience. We aimed to assess preferences for mode of delivery and reasons for preferences for CS in China to inform the development of future interventions to mitigate unnecessary CSs, which are those performed in the absence of medical indications.

**Methods and findings:**

We conducted a mixed-methods systematic review and included longitudinal, cross-sectional, and qualitative studies in mainland China, Hong Kong, and Taiwan that investigated preferences for mode of delivery among women and family members and health professionals, and the reasons underlying such preferences. We searched MEDLINE/PubMed, Embase, CINAHL, POPLINE, PsycINFO, Global Health Library, and one Chinese database (CNKI) using a combination of the key terms ‘caesarean section’, ‘preference’, ‘choice’, ‘knowledge’, ‘attitude’, ‘culture’, ‘non-clinical factors’, and ‘health professionals-patient relations’ between 1990 and 2018 without language restriction. Meta-analysis of quantitative studies and meta-synthesis of qualitative studies were applied. We included 66 studies in this analysis: 47 quantitative and 19 qualitative. For the index pregnancy, the pooled proportions of preference for CS reported by women in longitudinal studies were 14% in early or middle pregnancy (95% CI 12%–17%) and 21% in late pregnancy (95% CI 15%–26%). In cross-sectional studies, the proportions were 17% in early or middle pregnancy (95% CI 14%–20%), 22% in late pregnancy (95% CI 18%–25%), and 30% postpartum (95% CI 19%–40%). Women’s preferences for CS were found to rise as pregnancy progressed (preference change across longitudinal studies: mean difference 7%, 95% CI 1%–13%). One longitudinal study reported that the preference for CS among women’s partners increased from 8% in late pregnancy to 17% in the immediate postpartum period. In addition, 18 quantitative studies revealed that some pregnant women, ranging from 4% to 34%, did not have a straightforward preference for a mode of delivery, even in late pregnancy. The qualitative meta-synthesis found that women’s perceptions of CS as preferable were based on prioritising the baby’s and woman’s health and appeared to intensify through interactions with the health system. Women valued the convenience of bypassing labour because of fear of pain, antagonistic relations with providers, and beliefs of deteriorating quality of care during labour and vaginal birth, fostering the feeling that CS was the safest option. Health professionals’ preference for CS was influenced by financial drivers and malpractice fears. This review has some limitations, including high heterogeneity (despite subgroup and sensitivity analysis) in the quantitative analysis, and the potential for over-reporting of women’s preferences for CS in the qualitative synthesis (due to some included studies only including women who requested CS).

**Conclusions:**

Despite a minority of women expressing a preference for CS, individual, health system, and socio-cultural factors converge, contributing to a high CS rate in mainland China, Hong Kong, and Taiwan. In order to reduce unnecessary CSs, interventions need to address all these non-clinical factors and concerns.

**Systematic review registry:**

Prospero CRD42016036596.

## Abstract 2

In a systematic review of longitudinal, cross-sectional and qualitative studies, Qian Long and colleagues provide insight into the rates of medically non-indicated caesarean section in China.

## Author summary

**Why was this study done?:**

- In the last 3 decades, China has witnessed a dramatic increase in cesarean section (CS) rates. It has been argued that non-clinical factors, including women’s personal preferences, are driving the increase in CSs.
- Internationally, 1 systematic review and other types of reviews have studied women’s preference for CS, but they included few, if any, studies from China. We found only 1 existing narrative review of CS perspectives in China.
- To the best of our knowledge, to date, there has been no systematic review examining CS preference in China comprehensively, nor using a qualitative and quantitative approach synergistically to broaden the evidence base and improve our understanding of factors shaping preferences and how they interact.

**What did the researchers do and find?:**

- We conducted a mixed-methods systematic review and included longitudinal, cross-sectional, and qualitative studies in mainland China, Hong Kong, and Taiwan that investigated preferences for CS among women and family members and health professionals, and the reasons underlying such preferences.
- This review included 66 studies (47 quantitative and 19 qualitative studies). It shows a minority of women expressing a preference for CS, and that women’s preferences for CS are shaped by disincentivising healthcare system interactions, unsatisfactory relationships with providers, beliefs of deteriorating quality of care during labour and vaginal birth, and social support of the ‘right to choose’ CS.
- Fear of vaginal birth because of perceived risks to the baby is prevalent in quantitative studies and a paramount concern of women and clinicians in the qualitative studies, with the subsequent clinicians’ fear of litigation in the event of disappointing outcomes.

**What do these findings mean?:**

- Both the quantitative and qualitative findings in this review suggest that women’s request for CS on the basis of genuine preference for this mode of delivery is unlikely to be the major driver of high CS rates and that women’s preference may underscore health system deficiencies and suboptimal relationships with health professionals.
- The rate of unnecessary CSs—those performed in the absence of medical indications—is unlikely to reduce without multifaceted strategies targeting health professionals and healthcare systems, in addition to women.
- Formative research to understand facilitators of and barriers towards optimising the use CS is urgently needed to inform the development of contextualised multifaceted interventions to reduce unnecessary CSs.

## Introduction

In 2015, the World Health Organization released the *WHO Statement on Caesarean Section Rates*, which advises that population-level cesarean section (CS) rates higher than 10% are not associated with reductions in maternal and newborn mortality rates, while every effort should be made to provide CS to women in need [1–3]. CS, as any surgery, is associated with short-term and long-term risks to women and babies and has the potential to complicate future pregnancies [4]. Between 1990 and 2014, the global CS rate increased by 12.4%, with the highest absolute increase in Latin America and the Caribbean (19.4%) and the lowest in Africa (4.5%). In 2014, the estimated global CS rate was 18.6%, ranging from 7.3% in Africa to 40.5% in Latin American and the Caribbean [5].

One of the countries with the most dramatic increase in CS rates in recent decades is China. In mainland China, the CS rate at the population level increased from 3% in 1988 to 39% in 2008, with the most marked increase in urban areas [6]. A more recent study reported that the average CS rate was 34.9% in 2014 [7]. While the latter study reports that CS rates have recently declined in some urban areas, still half of all births in big cities occur by CS. A similar trend was found in Hong Kong and Taiwan, with CS rates reaching to 27.4% in Hong Kong in 1999 [8] and 35% in Taiwan in 2007 [9]. Non-clinical indications for CS have become important contributors to the increase [10,11]. For example, analysis of maternal and child health surveillance data (1994–2006) found that in southeast China the increase in CS rate after 1998 was almost entirely attributable to maternal request [12]. Other researchers have found that women in mainland China, Hong Kong, and Taiwan may request CS due to fear of labour pain, perceived safety of CS, and perceived better quality of life after birth [8,9,13].

Health professionals play an important role in decision-making processes in China [14–16]. In Hong Kong, elective CS is available and negotiable in private health facilities [8]. In Taiwan, both public and private health facilities perform elective CS, but CS without clinical indications is not covered by health insurance [9]. In mainland China, in the early 1980s, the national healthcare system started the implementation of an approach that focused on hospital-based, medicalized care, with a significant shift to 99.7% of all births occurring in health facilities by 2015 [17]. Too little is currently known about why and how China’s healthcare system, while endeavouring to ensure access to safe care for all women and babies, also experienced one of the highest observed rises in CS rates, far beyond that from which health benefits ensue.

In 2016, population policy in mainland China changed from allowing families to have 1 child to allowing them to have 2 children. This change has significant implications for national health service resources, including the use of CS to optimise maternal and infant health [18]. An in-depth understanding of the role of and reasons for women’s and healthcare providers’ preferences for mode of delivery in China is paramount to inform relevant policy and intervention strategy development aiming to mitigate unnecessary CSs, which are those performed in the absence of medical indications. We conducted a mixed-methods systematic review to assess women’s, family members’, and health professionals’ preferences for mode of delivery in mainland China, Hong Kong, and Taiwan and to map the reasons for preferences for CS, including societal, cultural, individual, and health system factors.

## Methods

### Search strategy and selection criteria

This mixed-methods review is part of a global review of women’s and healthcare providers’ preference for CS. The protocol was registered in PROSPERO (registration number: CRD42016036596), and this review was conducted according to the pre-specified analysis plan (S1 Text). We included original studies conducted in mainland China, Hong Kong, and Taiwan that investigated preferences of women and family members and healthcare professionals for mode of delivery, and the reasons underlying such preferences. No specific type of population was excluded. We included quantitative, qualitative, and mixed-methods studies. For the quantitative component, data from longitudinal and cross-sectional studies as well as baseline data from interventional studies were eligible for inclusion. For the qualitative component, studies were eligible for inclusion if the qualitative methods of data collection and analysis were explicitly reported. Studies that did not report on the methods used for data collection and analysis, or were based on secondary data analysis, were excluded.

The following databases were systematically searched: MEDLINE/PubMed, Embase, CINAHL, POPLINE, PsycINFO, Global Health Library, and one Chinese database CNKI (China Knowledge Resource Integrated Database), using a combination of the key terms ‘caesarean section’, ‘preference’, ‘choice’, ‘knowledge’, ‘attitude’, ‘culture’, ‘non-medical factors’, and ‘health professionals-patient relations’ between 1990 and 2016 without language restrictions. The search was updated on 18 April 2018 for all English databases to include studies published since the initial search. We added a couple of Chinese terms suggested by a peer reviewer and updated the Chinese database search on 10 May 2018. The search strategy for each database is presented in S1 Table. In addition, the reference lists of included studies were screened to identify additional relevant studies. All citations identified through the electronic databases were downloaded into EndNote, and duplicates deleted. Two reviewers independently screened titles and abstracts to select potentially relevant citations. When a citation was considered relevant or when the title/abstract was deemed insufficient for deciding inclusion/exclusion, the full texts were retrieved and evaluated. Discrepancies and uncertainties at any stage in this selection process were resolved through discussion with a third reviewer until consensus.

Potentially relevant citations were read and assessed and information extracted using a standardised form specifically designed for this review. Data were extracted by one reviewer and checked by a second reviewer. Disagreements were discussed and resolved through consensus. The information extracted included characteristics of the study, methods, and population as well as the relevant outcomes (preference for and reasons for preference for mode of delivery and opinions related to different modes of delivery). For the quantitative component, numerical data (numbers or percentages) were extracted. For the qualitative component, themes, authors’ interpretations, and participants’ quotations were extracted. S2 Table shows the data extraction form. When the data in the original publications were not sufficiently detailed or were unclear, we contacted the authors for clarification and additional information.

### Quality assessment of included studies

Methodological quality and transparent reporting of quantitative studies and of the quantitative components from mixed-methods studies that met the inclusion criteria were assessed using 10 quality criteria developed by the review group on the basis of existing instruments for observational studies (STROBE, NEWCASTLE, and Circum Network’s *Assessing Survey Research*) [19–21]. The 10 questions evaluate the reliability and quality of the information by assessing the eligibility criteria, sample size, representativeness, response rate, clarity of the questions/statements, ethical considerations, clarity of data (including numerators, denominators, and missing values), and consistency between the research question and data reported (S2 Text). Each question was scored with 1 point. The alternatives for each question were scored as ‘not reported’ or unsatisfactory (0 points) or satisfactory (1 point). Four authors (QL, FY, APB, and MDR) assessed the quality of all included quantitative studies independently and reached consensus through discussion in the case of discrepancies. Each study could be graded from 0 to a maximum score of 10, and the median score across all included studies was calculated. We considered the overall quality of a study to be ‘low’, ‘middle’, or ‘high’ if the grade was lower than, the same as, or higher than the median score, respectively.

Qualitative studies and the qualitative components from mixed-methods studies that met the inclusion criteria were assessed by 3 authors (QL, CK, and FY). Quality appraisal was carried out according to a checklist described by Walsh and Downe [22], and articles were graded according to Downe and Simpson [23]. A grade of A was assigned to papers that had no or few flaws, and a grade of D represented studies with significant flaws that could threaten the credibility of the papers. Any differences in the authors’ appraisals were resolved between the 2 native-Chinese-speaking authors (QL and FY) and another native-Chinese-speaking social scientist for the Chinese language papers and 2 authors (QL and CK) for the papers published in English (S3 Table).

The final grading of both quantitative and qualitative studies is listed in S4 Table.

### Data analysis

Quantitative studies that reported prevalence of and reasons for preferring CS were included in the analysis. The planned dummy tables for the analysis are presented in S1 Text. A meta-analysis of proportions of preference for CS was conducted. The pooled proportion was calculated as the Freeman–Tukey variant of the arcsine square root of transformed proportion, using inverse variance weights for the random effects model [24]. The subgroup analysis was conducted with stratification of longitudinal and cross-sectional studies based on the time when the preference was reported (early or middle pregnancy [second trimester], late pregnancy [third trimester], or during the postpartum period) and parity (nulliparous or multiparous, if specified in the studies). Across longitudinal studies, a mean difference was used to examine change of preference for CS over the pregnancy. Review Manager version 5.3 (RevMan; Cochrane Community, Oxford, UK) was used to perform these analyses. Significant heterogeneity was tested for (*I*^2^ > 40%), and when heterogeneity could not be explained by subgroup analysis and sensitivity analysis, the meta-regression analysis was performed using Comprehensive Meta-Analysis software adjusting for study region (Hong Kong and Taiwan, and mainland China divided into eastern, central, and western areas), location (urban, rural, or mixed), level of study health facilities (primary and secondary, tertiary, or mixed), risk for the pregnancy as defined by the study authors (low risk or not specified), and quality of study (low, middle, or high). Reasons for preferring CS reported by women were mapped and grouped into several categories and were summarised as a brief narrative.

The qualitative meta-synthesis method used was based on the interpretive meta-ethnography approach [25]. S1 Box presents a summary of the qualitative synthesis process. Ten of the included studies in Chinese were translated into English by a native Chinese speaker (QL) and a native English speaker (CK), to ensure that the meaning from the original text was preserved as accurately as possible. Key themes, categories, metaphors, phrases, ideas, and concepts were then extracted. This was done at the level of author interpretation and from quotations from participants reported in the results section of each study, beginning with the earliest paper [26]. This process was repeated for all included qualitative studies to generate a list of codes from which 26 initial concepts were developed. Two authors independently identified and tabulated codes, initial concepts, and emergent themes, before coming together to explore similarities, inconsistencies, and contradictions in the data (CK and QL). From this, 4 final themes and a line of argument synthesis were constructed. Confidence in findings constructed by the synthesis process was assessed using the GRADE-CERQual approach [27–32].

This systematic review is reported following the PRISMA statement guidelines for reporting systematic reviews [33] and the ENTREQ statement guidelines to enhance transparency in reporting qualitative evidence synthesis [34].

## Results

**Fig 1:**
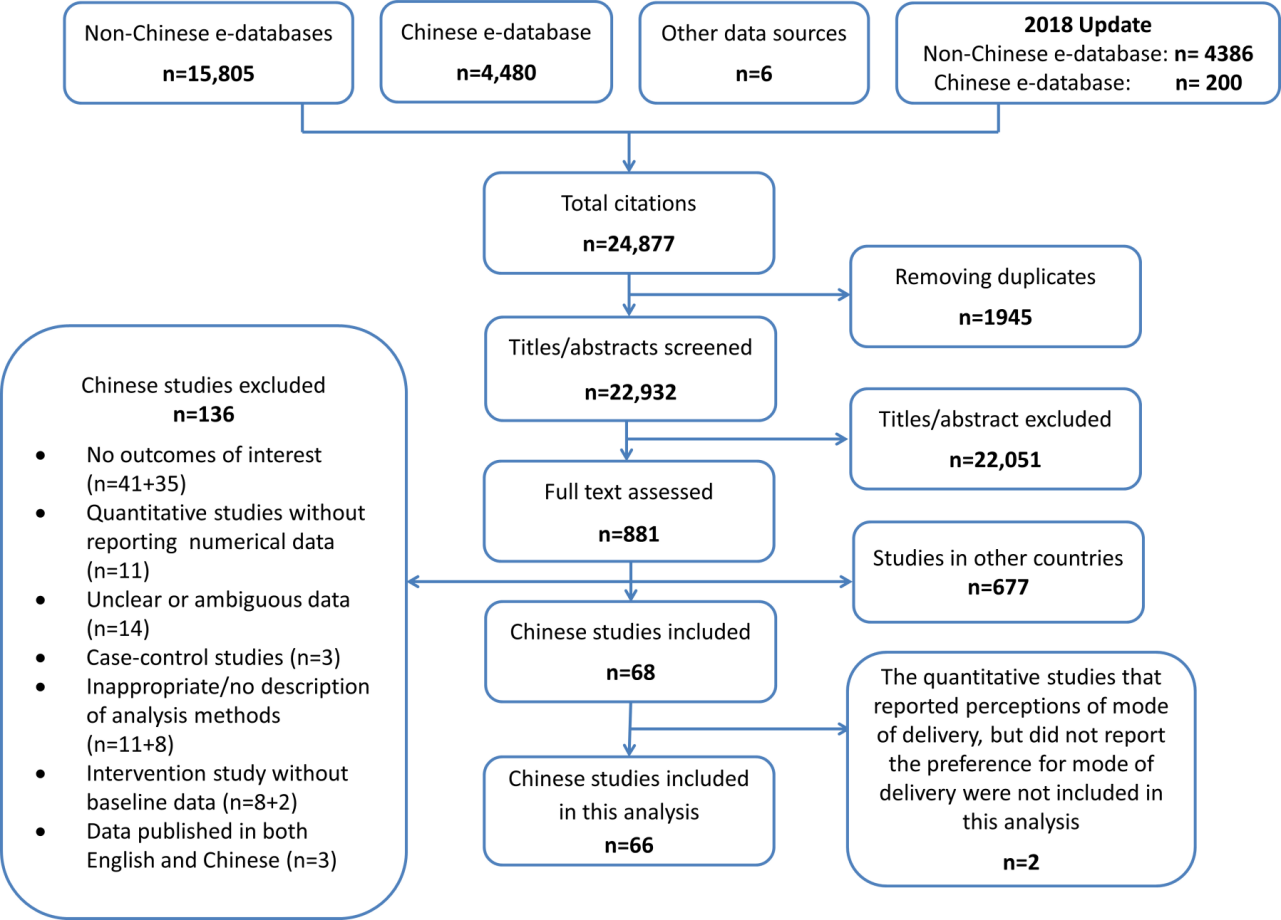
Flow chart of the study identification and selection.

The initial search strategy for the global review yielded 19,299 unique citations, and the updated searches yielded an additional 3,633 citations, in total 22,932 citations. After assessing titles and abstracts, 22,051 citations were excluded and 881 potentially relevant studies were retrieved for full text assessment. Of these 881 studies, 204 studies were conducted in China, of which 136 were excluded mainly because they did not report a relevant outcome or because of inappropriate study design and/or analysis methods. The review profile is presented in Fig 1.

A total of 66 studies (21 in English and 45 in Chinese) were included in the analysis [8,9,14,15,26,35–95]. The main characteristics of the studies included are presented in Table 1 (more detailed information for each study is presented in S4 Table). There were 47 quantitative [8,9,14,15,35–77] and 19 qualitative studies [26,78–95]. Most studies were conducted in urban areas, 58 in mainland China, and 8 in Hong Kong or Taiwan. All quantitative studies and most qualitative studies were facility-based. Sixty-four studies recruited women of childbearing age; 25 studies involved nulliparous women, 1 involved multiparous women, 15 involved both nulliparous and multiparous women, and 17 did not report parity. Six studies recruited women with previous CS. Four studies involved pregnant women’s family members, and 10 studies included healthcare professionals. Our quality assessment identified 14 quantitative and 5 qualitative studies as low quality.

**Table 1:** Summary of characteristics of included studies.

Characteristic	Number of studies	Studies
**Total**	66	[8,9,14,15,26,35–95]
**Language**		
Chinese	45	[35,37,38,40–67,74–84,87,89,90]
English	21	[8,9,14,15,26,36,39,68–73,85,86,88,91–95]
**Year of study**		
2000–2010	19	[8,9,15,35,37,39,43,45–47,51,52,74,78,81,85,86,88,95]
2011 or later	40	[14,36,38,40,41,42,44,48,50,53–73,75–77,82,83,89–92,94]
Not specified	7	[26,49,79,80,84,87,93]
**Study design**		
Quantitative study	47	[8,9,14,15,35–77]
Longitudinal	8	[9,14,15,35,36,68,69,75]
Cross-sectional	35	[8,37,39,42–54,56–67,70–74,76,77]
Experiment (baseline)	4	[38,40,41,55]
Qualitative study	19	[26,78–95]
**Study region**		
Mainland China	58	[14,15,35–38,40–72,74–84,86–92,94]
East	35	[14,15,35–38,40,44,46–49,52–54,56,57,59,60,63,65,69,70,74–76,78,79,82,87,89,90–92,94]
Central	15	[41,42,50,51,55,58,61,62,64,71,77,81,84,86,88]
West	5	[45,66–68,83]
Across regions	2	[72,80]
Unknown	1	[43]
Hong Kong and Taiwan	8	[8,9,26,39,73,85,93,95]
**Location**		
Urban	51	[8,9,14,15,26,35–39,41,44–49,51–54,56–60,62–64,66–70,73–80,82–84,87,89–91,94]
Rural	6	[40,42,65,81,86,88]
Mixed	5	[50,55,61,71,92]
Unknown	4	[43,72,93,95]
**Population**		
Facility-based	62	[8,9,14,15,26,35–79,82–84,87–95]
Population-based	1	[86]
Mixed	3	[80,81,85]
**Participants**		
Women	64	[8,9,14,15,26,35–73,75–83,85–95]
Nulliparous	25	[8,14,15,26,36,40–42,44–47,49,53,62,65,68,69,78,79,82,83,85,87,94]
Multiparous	1	[66]
Nulliparous and multiparous	15	[9,39,56,57,60,61,63,64,67,70–72,88,91,92]
Women with previous CS	6	[75–77,90,93,95]
Unknown	17	[35,37,38,43,48,50,51,52,54,55,58,59,73,80,81,86,89]
Family members	4	[36,50,61,81]
Healthcare professionals	10	[35,74,80,81,84,86,90–93]
**Quality of included studies**		
Low	19	[37,38,40,41,44–46,48,52,54,58,60,62,74,78–80,83,89]
Middle	18	[49,51,56,57,59,61,64,67,72,76,77,81,82,84–87,90]
High	29	[8,9,14,15,26,35,36,39,42,43,47,50,53,55,63,65,66,68–71,73,75,88,91–95]

### Quantitative synthesis

#### Prevalence of preference for CS

**Fig 2:**
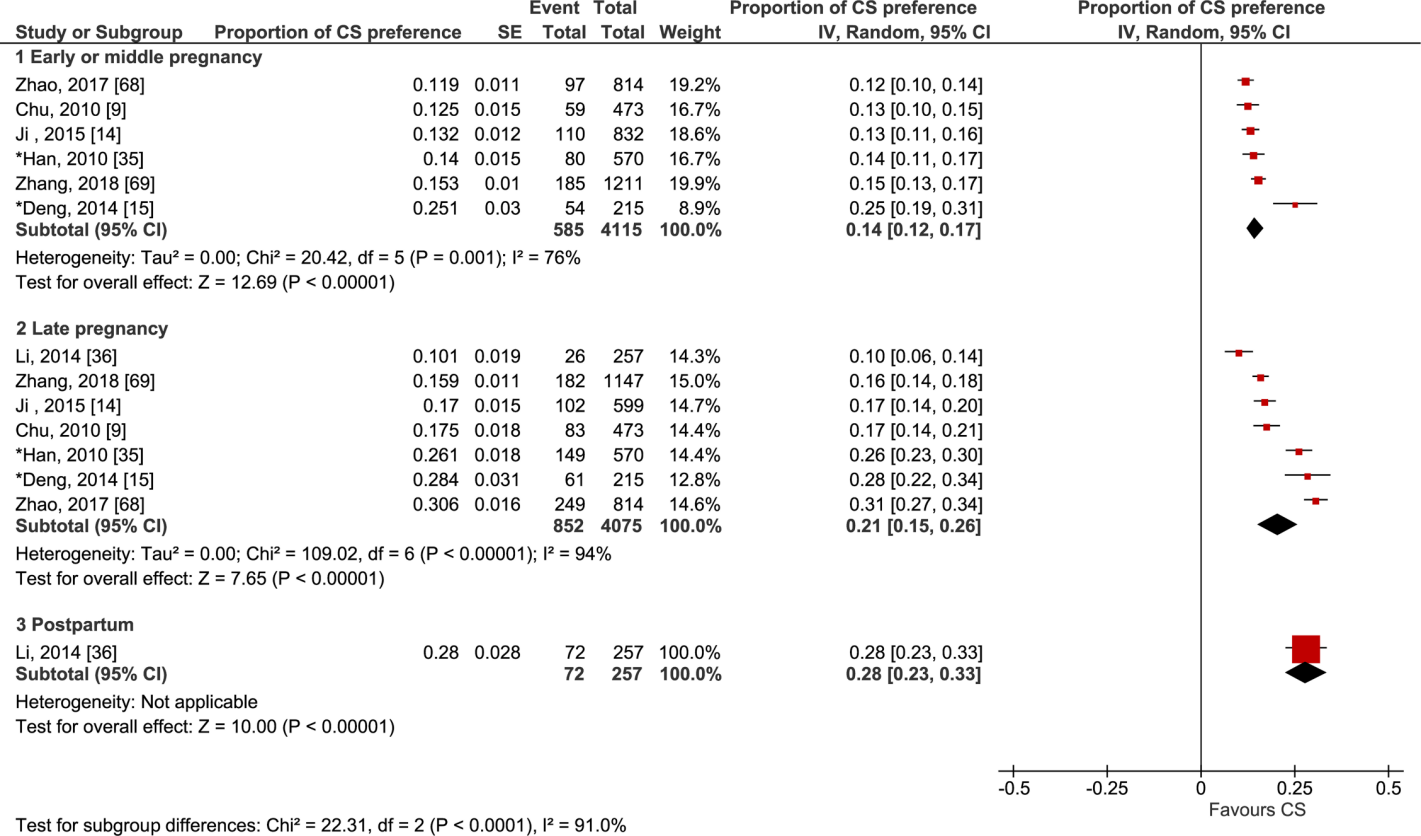
Forest plot of the proportion of women preferring cesarean section by time of reporting the preference: longitudinal studies. *Retrospective design, in which women were asked to recall their preference for a mode of delivery in early or middle pregnancy and in late pregnancy. CS, cesarean section; IV, inverse variance.

**Fig 3:**
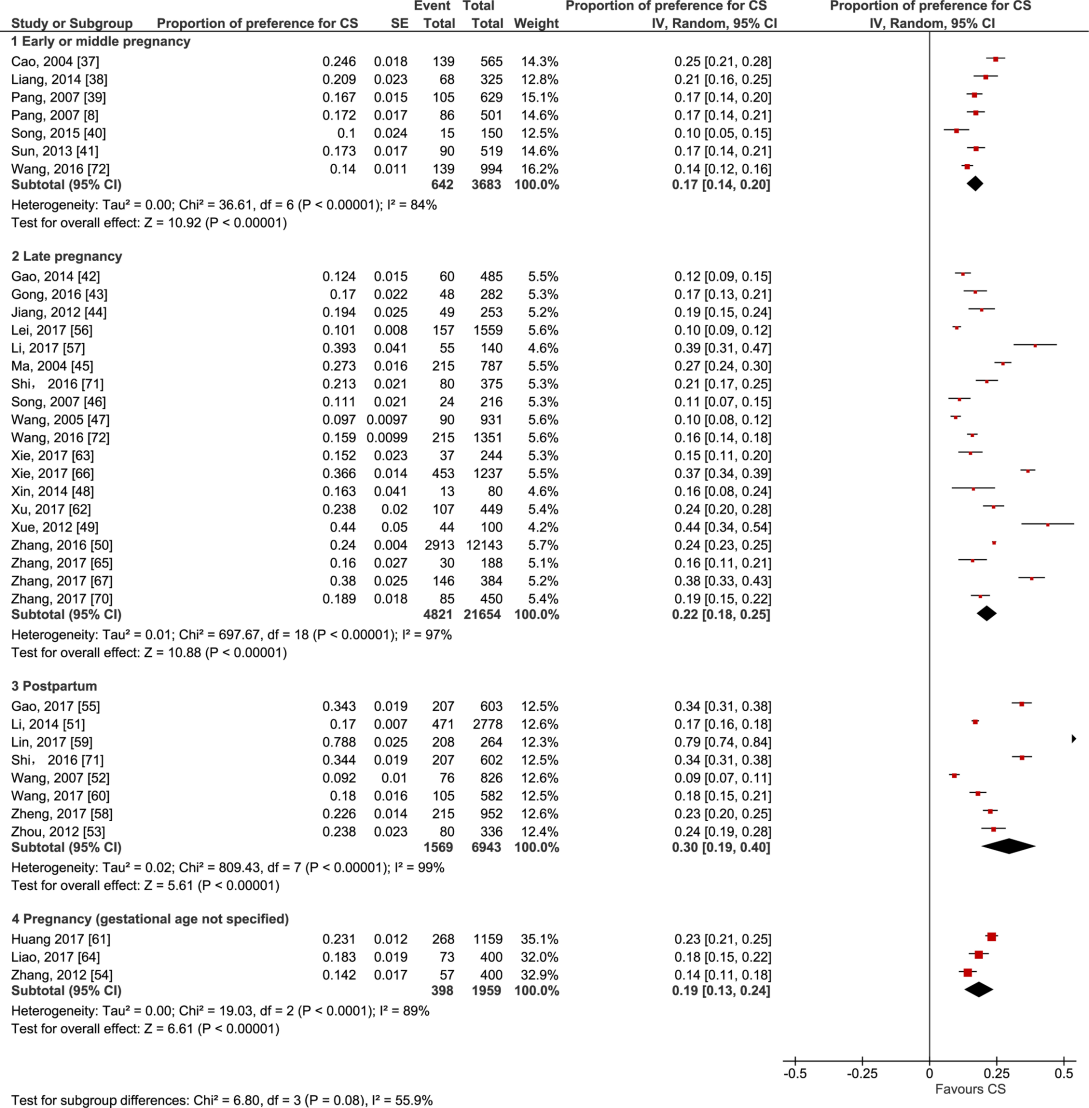
Forest plot of the proportion of women preferring cesarean section by time of reporting the preference: cross-sectional studies. CS, cesarean section; IV, inverse variance.

Forty-two studies investigated women’s preference for CS for their current pregnancy or for their recent delivery (when preference was reported after birth) [8,9,14,15,35–72]. Figs 2 and 3 show forest plots of the proportion of women preferring CS by when the preference was reported by the woman (i.e., early or middle pregnancy, late pregnancy, or postpartum) across longitudinal studies and cross-sectional studies, respectively.

In the stratification of the study design, the pooled proportions of preference for CS reported by women in longitudinal studies were 14% in early or middle pregnancy (95% CI 12%–17%; χ^2^ = 20.42; df = 5 [*p <* 0.01]; *I*^2^ = 76%) [9,14,15,35,68,69] and 21% in late pregnancy (95% CI 15%–26%; χ^2^ = 109.02; df = 6 [*p <* 0.01]; *I*^2^ = 94%) [9,14,15,35,36,68,69]. In cross-sectional studies, the proportions were 17% in early or middle pregnancy (95% CI 14%–20%; χ^2^ = 36.61; df = 6 [*p <* 0.01]; *I*^2^ = 84%) [8,37–41,72], 22% in late pregnancy (95% CI 18%–25%; χ^2^ = 697.67; df = 18 [*p <* 0.01]; *I*^2^ = 97%) [42–50,56,57,62,63,65–67,70–72], and 30% postpartum for the index pregnancy (95% CI 19%–40%; χ^2^ = 809.43; df = 7 [*p <* 0.01]; *I*^2^ = 99%) [51–53,55,58–60,71]. More multiparous women reported preference for CS (33%, 95% CI 31%–35%; χ^2^ = 114.78; df = 3 [*p <* 0.01]; *I*^2^ = 97%) [56,66,67,72] than nulliparous women (13%, 95% CI 12%–14%; χ^2^ = 282.46; df = 15 [*p <* 0.01]; *I*^2^ = 95%) [8,39–42,44–47,49,53,56,62,65,67,72]. Both univariate and multivariate meta-regressions showed significant association of the prevalence of preference for CS with parity (*p <* 0.05), but there was no significant association with study design, time preference was reported, study region, location, level of study facilities, risk for the pregnancy as defined by the study authors, and quality of study as assessed by the review authors. These results are presented in S1 Data. Moreover, 18 of 42 studies (4 longitudinal and 14 cross-sectional studies) [9,14,15,43–49,54,56,57,60,63,64,69,72] revealed that some pregnant women, ranging from 3% to 34%, did not have a straightforward preference for a mode of delivery, even in late pregnancy (S5 Table).

Six longitudinal studies [9,14,15,35,68,69] investigated the change of preference from early or middle pregnancy to late pregnancy, whether prospectively or retrospectively. All 6 studies reported an increase in the preference for CS as birth time approached, and the mean difference to test the change was statistically significant (mean difference 7%, 95% CI 1%–13%; χ^2^ = 63.76; df = 5 [*p <* 0.01]; *I*^2^ = 92%). One longitudinal study in mainland China investigated both women’s and their partners’ preferences for mode of delivery in late pregnancy and immediately postpartum if they could hypothetically choose again [36]. In this study, while only 10% of women reported a preference for CS in late pregnancy, this preference rose to 28% after giving birth. Likewise, the preference for CS among their partners also increased from 8% in late pregnancy to 17% in the immediate postpartum period.

One study reported that 23% (73 of 319) of Chinese women who were either pregnant or had given birth within the past 3 years in Hong Kong would prefer CS if financial and clinical factors were not considered [73]. One study in eastern China investigated maternity care providers’ preference for mode of delivery if they or their partners were pregnant, and found that one-fifth of maternity care providers (94 of 462) preferred CS [74].

Three studies (1 longitudinal and 2 cross-sectional studies) in mainland China investigated the preference for CS of pregnant women with previous CS [75–77]. In the longitudinal study, 90.2% of women preferred CS in early pregnancy, and the proportion of preference for CS showed moderate decrease in mid-term pregnancy (77.3%) and late pregnancy (71.1%) [75]. Two cross-sectional studies reported 48.7% and 44.6% of women preferring CS when they had prenatal visits for the current pregnancy [76,77].

#### Reasons for preferring CS

Fourteen quantitative studies reported reasons for women’s preference for CS (S6 Table) [8,36,37,44,46,50,54,55,60–63,70,73], which were summarised into 5 categories (Table 2). Across studies, the 3 most common reasons underlying the preference for CS were pain-related fear of vaginal birth (with the proportion of women giving this reason ranging from 8.1% to 80.6%) [8,36,37,44,46,50,60–63,70,73], the perceived maternal short-term risks of vaginal delivery (e.g., fear of perineal cut or tearing of perineum) (3.8% to 59.0%) [8,36,44,46,55,60–63,70,73], and the perceived risks of vaginal delivery for the baby (7.5% to 54.5%) [8,36,37,46,50,54,55,63,70,73]. Perceived maternal long-term risks of vaginal delivery (e.g., sexual dissatisfaction) was reported as a reason for preferring CS in 7 studies, with the proportion of women giving this reason ranging from 1.4% to 20.8% [36,44,46,61,62,70,73]. Regarding cultural and societal beliefs, choosing an auspicious date for a birth was reported in 7 studies, with the proportion of women giving this reason ranging from 1.1% to 19.2% [44,46,50,61,62,70,73]. In 5 studies the convenience of planning the birth was reported as a reason for preferring CS (5.8% to 64.8%) [8,36,60,63,73]. In addition, in 2 studies a few women reported choosing CS because of doctor’s or midwife’s advice [8,36], and in another 4 studies women reported choosing CS because of potential clinical indications (29.9% to 50.9%) [50,55,61,73], although 2 of the studies recruited low-risk pregnant women [50,55]. One study reported the reason of choosing CS due to prior CS (17.6%) [70].

**Table 2:** Reasons for CS preference reported by women: quantitative surveys.

Study	Pain-related fear of vaginal birth	Fear of perceived risk of vaginal delivery			Convenience of planning	Cultural and societal beliefs^c^	Medical indications for CS and other reasons
		Perceived maternal short-term risks^a^	Perceived maternal long-term risks^b^	Perceived risks for the baby			
Cao, 2004 [37]	42.1% (59)	NR	NR	17.1% (24)	NR	NR	NR
Pang, 2007 [8]	25.0% (13)	3.8% (2)	NR	44.0% (23)	5.8% (3)	NR	5.8% (3)
Song, 2007 [46]	70.8% (17)	33.3% (8)	20.8% (5)	45.8% (11)	NR	12.5% (3)	NR
Jiang, 2012 [44]	48.9% (24)	14.3% (7)	4.1% (2)	NR	NR	8.2% (4)	NR
Zhang, 2012 [54]	NR	NR	NR	54.5% (31)	NR	NR	NR
Li, 2014 [36]	80.6% (58)	25.0% (18)	1.4% (1)	18.1% (13)	11.1% (8)	NR	6.9% (5)
Loke, 2015 [73]*	79.5% (58)	26.0% (19)	16.4% (12)	53.4% (39)	27.4% (20)	19.2% (14)	45.2% (33)
Zhang, 2016 [50]	27.5% (802)	NR	NR	7.5% (219)	NR	3.5% (103)	50.9% (1483)
Gao, 2017 [55]	NR	36.8% (74)	NR	37.8% (76)	NR	NR	39.3% (79)
Huang, 2017 [61]	35.1% (94)	16.8% (45)	4.9% (13)	NR	NR	1.1% (3)	29.9% (80)
Xu, 2017 [62]	47.7% (51)	25.2% (27)	9.3% (10)	NR	NR	7.5% (8)	NR
Xie, 2017 [63]	8.1% (3)	45.9% (17)	NR	13.5% (5)	29.7% (11)	NR	NR
Wang, 2017 [60]	68.6% (72)	59.0% (62)	NR	NR	64.8% (68)	NR	NR
Zhang, 2017 [70]	16.5% (14)	23.5% (20)	9.4% (8)	41.2% (35)	NR	10.6% (9)	17.6% (15)

Data given as percent (*n*).

^a^Perceived maternal short-term risks included perineal cut, perineum tears, perineal trauma, and trauma in general, and perceived safer or better recovery with CS.

^b^Perceived maternal long-term risks included impact on sexual life or anal/urinary incontinence or body-image-related concerns.

^c^Cultural and societal beliefs included choosing an auspicious date for a birth, i.e., one that was perceived as good fortune for the baby and family in the future, and women’s right to choose mode of delivery.

*This study investigated reasons for preference for CS among women who were either pregnant or had given birth within the past 3 years.

CS, cesarean section; NR, not reported.

### Qualitative synthesis

Of 19 qualitative studies (S4 Table), 15 were from mainland China [78–84,86–92,94], 1 from Hong Kong [26], and 3 from Taiwan [85,93,95]. Fourteen studies detailed the views of women [26,78–83,85–89,94,95]; 8 also explored the views of healthcare providers [80,81,84,86,90–93]. One study reported the views of only policy-makers and managers [84]; another study involved healthcare providers who had a CS for non-clinical reasons themselves [87]. The earliest included study was published in 2001 [26], the most recent in 2018 [93,94].

#### Description of themes

Meta-synthesis generated 10 emerging themes and 4 final themes: ‘beliefs about cesarean section’, ‘influence of healthcare system factors’, ‘societal context and social change’, and ‘women’s experiences’. Table 3 presents the summary of qualitative review findings and CERQual assessments. S7 Table summarises initial concepts, emergent themes, final themes, and supporting quotes. The qualitative data offer insight into why women may express a preference for, or actually have, a CS, and show how childbirth culture can change over time.

**Table 3:** Summary of qualitative review findings.

Summary of review finding	Studies contributing to the review finding	CERQual assessment of confidence in the evidence	Explanation of CERQual assessment
**Theme 1: Beliefs about CS**			
**Belief CS is now a safe/safer option for birth** Health professionals, health service personnel, and women reported believing CS to be a safe, accessible alternative to vaginal birth in China now. Strength of belief was related to availability and accessibility of the operation. Where CS was most common, women reported it no longer necessary to suffer the ‘twice pain’ of labour ending in an emergency CS.	19 studies—[26,78–95]	High confidence	All included 19 studies across different geographical regions of mainland China (largely from developed areas), Hong Kong, and Taiwan contributed to this finding. Most studies were conducted in urban settings, and the 3 studies in rural settings were in the same province. Overall, there were moderate methodological limitations and minor concerns on coherence, relevance, and adequacy.
**Belief CS has social and cultural advantages for birth** Women reported CS for non-medical reasons as a socially and culturally advantageous means to exert control over the process, timing, and physical consequences of birth. CS was favoured because of the cultural significance of women participating in decision-making, the social advantages of scheduling birth, and perceptions of a more dignified birth experience and longer-term preservation of pre-pregnancy sexual attractiveness.	13 studies—[26,78–80,82,84–87,89,92–94]	Moderate confidence	A total of 13 studies across different geographical regions of mainland China (largely from developed areas), Hong Kong, and Taiwan contributed to this finding, with moderate concerns on methodological limitations, coherence, and adequacy and minor concerns on relevance. Only 1 study in a rural setting contributed to this finding.
**Theme 2: Healthcare system factors**			
**Financial drivers, financial means, and financial burdens and CS** In mainland China, health professionals and health service personnel reported that CS provides a means of generating revenue for the healthcare system. Some healthcare professionals expressed concerns that financial incentives were given more precedence than clinical guidelines. Health professionals, women, and family members reported that CS had become affordable with socio-economic development and the implementation of supportive policy to address financial risk, particularly in rural areas (e.g., rural health insurance scheme [New Cooperative Medical Scheme]). Some health professionals and women in rural areas reported that CS still could be a financial burden for them and their families. In Taiwan, women indicated that elective CS would cause a heavy financial burden because both public and private health insurance did not cover elective CS. Women who had the financial means to afford CS reported more freedom to choose it. Only 1 recent study from mainland China discussed financial penalties incurred by hospitals with high CS rates (>40%).	10 studies—[26,81,82,84–88,91,93]	Moderate confidence	A total of 10 studies across different geographical regions of mainland China, Hong Kong, and Taiwan contributed to this finding, with moderate concerns on methodological limitations, coherence, and adequacy and minor concerns on relevance. Two studies recruited women who could freely choose CS and indicated that CS was affordable for them. Another study only briefly indicated that doctors would earn more money, with this finding not integral to themes.
**Mistrust between women and healthcare professionals** In mainland China, healthcare professionals and health service personnel expressed concerns about the lack of trust in their relationships with women and families, identifying the threat of *yi nao* (fear of recrimination) as driving their preference for CS for self-protection. Women expressed that hospital services were not patient-centred care, identifying lack of health service personnel’s support during labour and having no choice or having insufficient information to make a decision.	8 studies—[79–84,88,92]	Moderate confidence	This finding emerged in mainland China. A total of 8 studies contributed to this finding. Women’s views in relation to ‘non-patient-centred care’ supported this finding, but data were limited. Overall, there were minor concerns on relevance and moderate concerns on methodological limitations, coherence, and adequacy.
**Quality of care: Health professionals’ training, skills, experience, and influence** In mainland China, healthcare professionals and health service personnel were critical of the lack of skills and training available for vaginal birth as a consequence of the increasing use of CS and obstetric technology in urban and rural areas of China. Some professionals expressed concern about how little value was placed on midwifery as a profession and midwifery skills and training. In urban areas, women who chose CS expressed their worries caused by fetal monitoring, although the monitoring results did not indicate performing CS necessarily. Moreover, some health service personnel’s preference on giving birth by CS also impacted women’s choice. In rural areas, women thought the environment in high-level hospitals was more comfortable than that in primary health facilities. In township health centres, women valued midwives’ skills, whereas opinions on doctors’ competencies in vaginal birth in this setting varied. Recent investment in mainland China in midwifery care as part of the strategies to reduce unnecessary CS and promote primary vaginal birth and vaginal birth after CS (VBAC) may have improved perceptions of maternity care and the experiences of some women. In Taiwan, obstetricians’ recommendations were key factors influencing women’s decision on VBAC.	13 studies—[81–84,86–88,90–95]	High confidence	This finding was revealed in mainland China and Taiwan. A total of 13 studies contributed to this finding, with moderate concerns on methodological limitations, and minor concerns on coherence, adequacy, and relevance.
**Quality of care: Availability of labour support and pain relief during vaginal birth** The extent to which pain relief and/or support during labour and vaginal birth was available to women varied across studies. Some women reported a preference for CS because they felt unprepared to endure the pain of labour. Women who expressed a preference for CS feared little or no support, poor care, and the pain associated with labour and birth (including episiotomy without pain relief). Access to pain relief during and following CS was routine (and included general anaesthesia for elective cesareans). Where support and/or pain relief was available during labour and vaginal birth, women reported confidence in their ability to cope with labour pain, expressed their gratitude to health professionals for empowering them to deliver vaginally, and had less pain following childbirth.	11 studies—[26,79,81–83,86–89,91,93]	Moderate confidence	Eleven studies across different geographical regions of mainland China (largely from developed areas), Hong Kong, and Taiwan contributed to this finding, with moderate concerns on methodological limitations and adequacy and minor concerns on relevance and coherence.
**Theme 3: Societal context and social change**			
**Complexity and autonomy: Women’s right to choose CS in China** Women, healthcare professionals, and health service personnel reported the decision-making processes associated with choice of CS for non-medical reasons as complex. Some women reported choosing CS because of deep-rooted fears, whereas other women’s reasons were more negotiable. Political and socio-economic developments in China between 1980 and 2010 meant that women increasingly expect to have the choice of CS available to them, even where it is not. Social and legislative change, coupled with unprecedented financial growth and increased availability of medicalized care, meant that some women had the determination and the means to exercise complete autonomy over their choice of CS. Other women felt a moral responsibility to choose CS, while some healthcare professionals felt vaginal birth to be the ‘correct choice’. Where decision-making by women was possible, they expressed concerns that their expectation of choice still had to be skilfully negotiated with healthcare professionals and family members (husbands, parents). Since 2010, policies in mainland China to reduce unnecessary CS may have limited some women’s choice.	16 studies—[26,79–89,91–94]	High confidence	A total of 16 studies across different geographical regions of mainland China (largely from developed areas in urban settings; 3 studies in rural settings in 1 province), Hong Kong, and Taiwan contributed to this finding. Overall, there were moderate methodological limitations and minor concerns on coherence, relevance, and adequacy.
**Safety of baby paramount concern of women and families** In Hong Kong, Taiwan, and mainland China, women expressed concerns for their baby’s safety as the paramount consideration in their choice of delivery method. For many women it was to be their only baby, because of delayed childbearing, problems conceiving, or legislation limiting family size. In included studies from mainland China conducted during the 1-child policy in urban areas, both women and health professionals reported the pressure they felt as a result of having only ‘one chance to get it right’ for the woman and child. Women were only children too, adding an additional layer of complexity to clinician’s and women’s concerns. Women’s preferences for CS or vaginal birth were informed by their knowledge of both the immediate risks associated with delivery methods and longer-term concerns with optimal child development. The gradual relaxation of the 1-child policy since 2012 and the existence of the universal 2-child policy since 2016 means that women’s preferences for CS may be decreasing as they now consider the implications of primary CS for subsequent pregnancies and family spacing.	15 studies—[26,78,79,81–85,87–89,92–95]	Moderate confidence	A total of 15 studies across different geographical regions of mainland China (largely from developed areas in urban settings; 2 studies in rural settings in 1 province), Hong Kong, and Taiwan contributed to this finding. Overall, there were moderate concerns on methodological limitations and adequacy and minor concerns on coherence and relevance.
**Theme 4: Women’s experience of labour, vaginal birth, and CS**			
**Women’s experiences: How childbirth is a fundamental human concern of individuals and society** Women reported how their individual characteristics (age, parity, size, stature, demur), concerns (deeply rooted fear of pain), and priorities (safety of baby, protection of perineum) influenced their personal preferences for birth mode in conjunction with everyday information exchanges about birth in society at large (family, friends, celebrities, popular culture, internet). In the most recent studies, women reported receiving more information about the benefits of vaginal birth (including VBAC) from healthcare providers.	15 studies—[26,78,79,81–83,85,87–90,92–95]	High confidence	A total of 15 studies across different geographical regions of mainland China (largely from developed areas in urban settings), Hong Kong, and Taiwan contributed to this finding, with moderate concerns on methodological limitations and minor concerns on coherence, relevance, and adequacy.
**Women’s experiences: Heterogeneity, uncertainty, and unresolved meaning surrounding birth** Women reported how other women may differ in their expectations and experiences of birth and each woman must make her own choice of birth method, weighing up the risks and benefits to her. Many women expressed doubts about which birth method was preferable, with uncertainty and contradictions in care and outcomes surrounding both. Women who experienced vaginal birth reported positive and negative experiences. Some women who had CSs focused entirely on the risks of vaginal birth and the benefits of CS, while others reported feelings of doubt, regret (baby born too soon, not trying to birth vaginally), and loss of experience (at moment of birth and postnatally when unable to lift baby or breastfeed).	18 studies—[26,78–85,87–95]	High confidence	A total of 18 studies across different geographical regions of mainland China (largely from developed areas in urban settings), Hong Kong, and Taiwan contributed to this finding, with moderate concerns on methodological limitations and minor concerns on coherence, relevance, and adequacy.

#### Beliefs about CS

This theme captures the shifting views of individual women, healthcare providers, and policy-makers about CS as a safe, or safer, alternative to labour and vaginal birth, across urban and rural settings, and healthcare facilities. Similar to the findings from the quantitative data, not all participants in the qualitative studies preferred CS, but many believed it could have physiological advantages, and few dismissed it as an option. This may help explain the ambivalence reported as ‘no preference’ in the quantitative data. In all 19 qualitative studies, women knew CS as an option now, with strength of beliefs about CS related to availability, accessibility, and prior experience of medical care. Where CS was most common, women reported that it was no longer necessary to suffer the ‘twice pain’ of labour ending in emergency CS (high confidence) [26,78–90,92–95]. Respondent’s beliefs about CS also encompassed CS as having social and cultural advantages over labour and vaginal birth [26,78–80,82,84–87,89,92–94] (moderate confidence), although only 1 study from rural China contributed to this review finding [86]. In some of the recent studies, there was evidence that women’s and health professionals’ beliefs may be shifting towards valuing CS per se less, and vaginal birth (including VBAC) more [90,92,93–95].

#### Influence of healthcare system factors

Theme 2 captures participants’ views of the financial drivers behind rising and abating CS rates, antagonistic relationships between women and health professionals, and perceptions of the quality of care during labour and vaginal birth, including concerns about the skills and competencies of health professionals and/or access to labour support or pain relief. This theme may help explain why women’s preferences can change as pregnancy progresses and go some way to explaining the discrepancy between women’s expressed preferences and actual birth mode. In 8 qualitative studies, all from mainland China, women expressed concerns that the system was not ‘woman-centred’ and health professionals feared recrimination in the event of a poor health outcome (also known as *yi nao*) (moderate confidence) [79–84,88,92]. The quantitative findings report women’s fear of pain as an important reason for expressing a preference for CS. Responses in 11 qualitative studies showed perceptions of pain and access to pain relief varied (moderate confidence) [26,79,81–83,86–89,91,93]. While some women reported a preference for CS because they felt unprepared to endure the pain of labour, other women feared no access to pain relief or labour support for vaginal birth.

#### Societal context and social change

The third theme captures the uniqueness of China’s societal context during the years in which many the studies were undertaken (2001–2015), and the emergent landscape of childbirth in the 2-child policy era (since 2016). It provides context as to why the safety of the baby was, and remains, the paramount concern of women, families, and healthcare professionals. In 15 studies from Hong Kong, Taiwan, and mainland China, women expressed concerns for their baby’s safety, as, for most, it was to be their only baby, because of delayed childbearing, problems conceiving, or legislation limiting family size (9 included studies were conducted during mainland China’s 1-child policy in urban areas) (moderate confidence) [26,78,79,81–85,87–89,92–95]. In mainland China, the 1-child policy also coincided with the reform of women’s rights with the introduction of the Maternal and Infant Health Care Law. In 16 of the 19 qualitative studies, women, healthcare professionals, and health service personnel reported the decision-making processes associated with choice of CS for non-medical reasons as complex and linked to the extension of women’s rights (high confidence) [26,79–89,91–94]. In the most recent studies, the effects of policies to reduce unnecessary CSs coincided with population policy reform that meant that health professionals and women had to consider the implications of primary CS on subsequent pregnancies.

#### Women’s experiences

The final theme captures the complexity and diversity of women’s experiences of decision-making and actual modes of delivery. In 15 studies, women reported how their individual characteristics (age, parity, size, stature, demeanor), concerns (deeply rooted fear of pain), and priorities (safety of baby, protection of perineum) influenced their personal preferences for birth mode in conjunction with everyday information exchanges about birth in society at large (family, friends, celebrities, popular culture, internet) (high confidence) [26,78,79,81–83,85,87–90,92–95]. In 18 of the 19 studies, women reported how they had to make their own choice of birth method. Many women expressed doubts about which birth method was preferable with uncertainty and contradictions in care and outcomes surrounding both vaginal delivery and CS (high confidence) [26,78–85,87–95]. In the final interpretive stage of the analysis, findings were combined to represent our interpretation, through a line of argument. This line of argument synthesis is presented in Box 1.

**Box 1. Line of argument synthesis**

The qualitative meta-synthesis reveals how individual, health system, and societal factors converge to shape perceptions of CS and influence actual births. In mainland China, the 1-child policy (1979–2016) spanned generations of participants in 10 studies. This period was important in making the safety of the woman and baby paramount for families and healthcare providers, with China’s population policy an unintended contributing factor to CS preferences and rates. While ancient Chinese cultural beliefs about auspicious dates reportedly favoured the scheduling that a planned CS can offer, women also came to value the perceived convenience of bypassing labour, believing it could allay their fears about vaginal birth (inadequate labour support, insufficient pain relief, perineal trauma, sexual dysfunction). At the same time, health professionals, driven by financial incentives and fears of malpractice, also favoured planned CSs over more unpredictable vaginal births. The healthcare development approach in mainland China, which initially focused on the provision of specialised medical care, was perceived as devaluing midwifery and vaginal birth, and making CS a safe, available, and accessible option. Simultaneously, China’s rapidly emerging economy afforded some women and families the financial means to access elective CS, and legislation governing entitlement to care gave all women the right to choose CS. The convergence of these factors helps us to comprehend the unprecedented rise in CS rates in China. Likewise, the convergence of these factors helps explain why women’s preferences can and do change throughout pregnancy, as they are influenced by interactions with the healthcare system. It also goes some way to accounting for why so many women’s expressed preferences differ from their actual birth mode. Some women who did not express a preference for a CS but still had a CS turned out to have positive birth experiences, but this was not the case for all women. Similarly, health professionals had both favourable and unfavourable perspectives of CS and its consequences. The most recent studies add weight to the argument that individual, health system, and societal factors converge to shape perceptions of CS and influence actual births. They suggest that CS rates can abate with multifaceted strategies targeting women, healthcare professionals, and healthcare systems to foster a cultural change. The 2-child population policy may have the unintended consequence of reducing CS further as values and preferences shift towards primary vaginal birth and VBAC. This line of argument also supports the quantitative findings that preferences for mode of delivery are fluid, with women prioritising their own health and the health of their babies when forming these preferences.

## Discussion

### Summary of findings

In this systematic review, quantitative meta-analysis showed that around one-fifth or less of Chinese women across mainland China, Hong Kong, and Taiwan expressed a preference for CS, with fear of childbirth being the most prevalent reason for this preference. The preference for CS appears to increase as pregnancy progresses. Both quantitative and qualitative studies in this review revealed that some pregnant women did not have a straightforward preference for a mode of delivery, even in late pregnancy. The qualitative meta-synthesis identified that perceptions of CS as preferable were based on prioritising the baby’s and woman’s health and appeared to intensify through interactions with the health system across mainland China, Hong Kong, and Taiwan. In addition, financial drivers and malpractice fears influenced preference for CS among health professionals.

### Interpretation

Over the past 3 decades, the CS rate has rapidly increased worldwide, and it has been argued that maternal request for CS is one of the major driving factors [96,97]. Mazzoni et al. conducted a systematic review and meta-analysis of 38 observational studies across Latin and North America, Europe, Asia, and Africa in 2011, and reported an overall pooled women’s preference for CS of 15.6% (95% CI 12.5% to 18.9%) [98], which is similar to that found in this review. In addition, Mazzoni et al. included pooled preferences stratified for multiparous women (17.5%, 95% CI 13.4% to 21.8%) and women with a previous CS (29.4%, 95% CI 24.4% to 34.8%) [98]. Most studies in our review recruited nulliparous women in mainland China and Hong Kong, and there was no statistically significant difference in the reported preference for CS by study region across mainland China and Hong Kong and Taiwan. However, recent studies enquiring about the preference of multiparous women in mainland China showed much higher preferences for CS in women with a previous CS than those found by Mazzoni et al., ranging from 50% to 80%.

The qualitative component of this review elicited the multiple social, cultural, and health system factors that impact on preference and actual mode of delivery in China. Our meta-synthesis found beliefs about CS to be linked to its increasing accessibility and acceptability. Consistent with previous studies in other countries [99,100], this review found that women’s preferences changed as pregnancy progressed, and ambivalence about birth mode was evident, with women who expressed no clear preference. Women’s perceptions of CS as preferable were shaped by wider social change in mainland China, Hong Kong, and Taiwan, including the new reproductive right to choose CS, availability of and willingness to pay for medicalized childbirth care, and population policy change in mainland China. We found that the preference is influenced by some health system factors unique to mainland China, including disincentivising healthcare system interactions, unsatisfactory relationships between women and health professionals, and beliefs of deteriorating quality of care during labour and vaginal birth. Concerns about the lack of labour support and pain relief measures for women having a vaginal birth may explain why pain-related fear was the principal concern reported by women in the quantitative studies. Fear of vaginal birth due to perceived risks to the baby was also found to be common in quantitative studies and a paramount concern of women and clinicians in the qualitative studies, with the subsequent clinicians’ fear of litigation in the event of disappointing outcomes.

The qualitative studies suggest that financial drivers influenced health professionals’ preference for CS, which is reported as a factor contributing to the increase of CS rate in previous studies in mainland China, Taiwan, and other middle- and low-income countries [16,101–103]. In mainland China, public hospitals heavily reply on fee-for-service reimbursement, and healthcare providers’ salaries are tied up with the revenue generated for the facility through a bonus system [104]. Taiwan’s health insurance system set a global budget for obstetric services; however, performing elective CSs still generates higher financial benefits for physicians than normal delivery [103]. This review revealed concerns that financial drivers were given more precedence than clinical guidelines for performing CS.

Reduction of unnecessary CSs has been a global concern. A Cochrane review included 16 studies to evaluate the effectiveness of non-clinical interventions for reducing unnecessary CSs [105]. The interventions assessed included prenatal education and support programmes, decision aids, audit and feedback, training health professionals, insurance reform, and legislative changes. This Cochrane review concluded that there was insufficient evidence that these interventions targeting either pregnant women or health professionals were effective [105]. In mainland China, some strategies at the national, provincial, and hospital level, such as education for pregnant women and health professionals, setting a targeted CS rate at the health facility level, and removing financial incentives, have been introduced over the past decade at varying regional and system levels [7]. Nevertheless, there is a lack of rigorous design and formal evaluation of these strategies’ effectiveness, although CS rate declined in some areas coinciding with these efforts [7].

Since 2016, when mainland China stepped into the new era of ‘2 children in a family’, the healthcare system has faced challenges meeting increasing demands for high-quality maternity care, particularly with increasing numbers of older pregnant women and women who have had a previous CS [18,106]. A recent study suggested that the change in population policy may have led to an increase in the need for CSs due to the change in obstetric risks, and repeat CS following previous CS may be a key concern [106]. VBAC is recommended internationally as a safe option for most women. Several qualitative studies conducted in European countries with high or low VBAC rates suggested that clinical expertise, health professionals’ support for pregnant women, and shared decision-making were more likely to contribute to successful VBAC [107–109]. The lack of qualified health professionals and the tense relationship between women and healthcare providers found in this review raise the question of the acceptability and effectiveness of VBAC to reduce CS in mainland China. In Taiwan, the level of health professionals’ support and health insurance coverage were important influences on the availability of VBAC.

### Strengths and limitations of the review

To the best of our knowledge, this is the first mixed-methods systematic review to apply meta-analysis of quantitative studies and meta-synthesis of qualitative studies to provide a comprehensive picture of the preference for CS and the motivations for it in China. This review included studies in Hong Kong and Taiwan and across different regions of mainland China published in English and Chinese, and captured views of women, family members, healthcare providers, and health administrators on the preference and reasons for CS, rather than only considering women’s preferences in isolation of these influencing factors. This review also has several limitations. In our meta-analysis of preference for CS, we stratified and adjusted for selected important variables, but heterogeneity remained high, although similar to that reported in a previous meta-analysis of women’s preference for CS [98]. Among the 19 qualitative studies, 9 included women who were planning to have, or had had, a CS [26,78–83,87,89], 6 of which included by design only women who had requested a CS for non-clinical reasons [26,79,82,83,87,89]. This means some studies may have over-reported women’s preference for CS, by their exclusion of women who did not request a CS. This review only identified and included 4 studies from Hong Kong and 4 studies from Taiwan, and thus we cannot accurately differentiate and draw independent conclusions for mainland China, Hong Kong, and Taiwan. In addition, the quality of some Chinese publications was appraised as low using reporting standards. The data presented in the Chinese language publications were generally thin, possibly due to the word limits in journals. We did not exclude any study based on the quality assessment, but considered it in both the meta-analysis and CERQual assessments.

### Implications for future research

The findings of this review suggest that women’s requests for CS on the basis of preference for this mode of delivery are unlikely to be the major driver of high CS rates and that women’s preference may underscore health system deficiencies and suboptimal relationships between women and health professionals. As such, unnecessary CSs are unlikely to reduce without multifaceted strategies targeting also health professionals and healthcare systems. In this context, but also considering the increasingly global concern on the overuse of CS, there is an urgent need to invest in research to improve our understanding of stakeholders’ views on mode of delivery and how they relate to quality of maternity care, and to identify local barriers and/or good practices embedded in health systems (e.g., health financing and payment methods, health workforce training, and career development) towards optimising the use of CS. These insights will be critical to inform the development of contextualised multifaceted interventions to reduce unnecessary CSs, which can be subsequently assessed in randomised controlled trials.

### Conclusion

This review revealed the impact of multiple individual, health system, and societal factors on the preference for CS as a mode of delivery in mainland China, Hong Kong, and Taiwan, and demonstrated that they worked in an interactive way. It also provides an illustrative case of the underlying mechanisms at play that go beyond China’s unique context. From 1980 onwards, the processes of remarkable social and economic development, urbanisation, globalisation, widespread medicalization of childbirth, and shifting beliefs about CS have coincided. These developments within mainland China, Hong Kong, and Taiwan parallel a wider shift in beliefs about the increasing safety of CS outside of China [110–112]. There is emerging evidence from mainland China that beliefs about CS may be shifting again as a consequence of the 2-child policy era and the local strategy to reduce unnecessary CSs. There has been a steady rise in CS rates in most high-income countries and a notable increase in some middle-income countries, such as Brazil, observed since 1990 [5]. When underuse and overuse of CS coexist across and within countries, CS should be performed when needed, clinically effective, affordable, equitable, and responsible in its use of resources.

## Supporting information

S1 Box Summary of qualitative synthesis process. (DOCX)

S1 Data Meta-analysis results. (DOCX)

S1 Table Search strategies. (DOCX)

S2 Table Data extraction form. (XLSX)

S3 Table Assessment of quality of included qualitative studies. (DOCX)

S4 Table Characteristics of the included studies. (DOCX)

S5 Table Uncertain preference for a mode of delivery for current pregnancy: quantitative surveys. (DOCX)

S6 Table Reasons for preference for CS reported by women: quantitative studies. (DOCX)

S7 Table Summary of initial concepts, emergent themes, final themes, and supporting quotes. (DOCX)

S1 Text Protocol and analysis plan. (PDF)

S2 Text Quality assessment prompts for quantitative studies. (DOCX)

## Abbreviations

CS: cesarean section
VBAC: vaginal birth after cesarean section
