# Supplementary material for: Prevalence of and reasons for women’s, family members’, and health professionals’ preferences for cesarean section in China: A mixed-methods systematic review
Source: PLoS Med. 2018 Oct 16;15(10):e1002672. doi: 10.1371/journal.pmed.1002672 (PMC6191094; doi:10.1371/journal.pmed.1002672)
Supplement: S1 Box — (DOCX) [file pmed.1002672.s001.docx]

**S1 Box Summary of qualitative synthesis process**

| The qualitative synthesis followed five steps.   1. **Familiarization and quality assessment**   Nineteen qualitative studies that met a basic quality threshold of using qualitative methods for both data collection and analysis were included. Key findings of the ten included studies published in Chinese were translated into English by the reviewers (QL, a native Chinese speaker and CK, a native English speaker) to ensure the meaning from the original text was preserved as accurately as possible. Quality appraisal of each study was carried out according to a checklist described by Walsh and Downe [22] to assess study scope and purpose, design, sampling strategy, analysis, interpretation, researcher reflexivity, ethical dimensions, relevance and transferability, and the A-D grading of Downe [23] was applied. A grade of A was allocated to papers which had no or few flaws and D represented studies with significant flaws that could threaten the credibility of the papers. Supplementary appendix 5 presents the assessment of quality of included qualitative studies.   1. **Data extraction**   The characteristics of included studies, author interpretation (themes, theories and metaphors) and verbatim text (participant quotes) were entered into a form designed for the purposes of this review. Please see Supplementary appendix 3: Data extraction form.   1. **Coding**   Codes were developed from extracted data, starting with the earliest paper [26]. Codes were refined through comparison with the findings from all included studies and agreed by consensus (CK and QL). All extracted data from all included studies were coded into initial concepts. Supplementary appendix 11 presents the summary of initial concepts, emergent themes, final themes and supporting quotes.     1. **Interpretative synthesis**   Initial codes were grouped into emergent themes, first by reciprocal analysis (confirmatory of similarities in data across studies) and then by refutational analysis (integration of disconfirmatory data into emergent themes). Emergent themes were then synthesized into Summary of Findings (SoFs) statements and final themes, which were then used to construct a Line of Argument statement.   1. **GRADE-CERQual – Confidence in the Evidence from Reviews of Qualitative Research**   GRADE-CERQual is an approach to assess the confidence in qualitative evidence synthesis findings [27]. Assessment is at the level of emergent themes/SoFs as reported above. CERQual assessment comprises of four domains: 1)Methodological limitations - The extent to which there are problems in the design or conduct of the primary studies that contributed evidence to a review finding; 2)Relevance - The extent to which the body of evidence from the primary studies supporting a review finding is applicable to the context (perspective or population, phenomenon of interest, setting) specified in the review question; 3)Coherence - The extent to which the review finding is well grounded in data from the contributing primary studies and provides a convincing explanation for the patterns found in these data; 4)Adequacy – The assessment of the degree of richness and quantity of data supporting a review finding. Please see table 3 in the paper for SoFs and CERQual assessments. |
| --- |
