# Supplementary material for: Prevalence of and reasons for women’s, family members’, and health professionals’ preferences for cesarean section in China: A mixed-methods systematic review
Source: PLoS Med. 2018 Oct 16;15(10):e1002672. doi: 10.1371/journal.pmed.1002672 (PMC6191094; doi:10.1371/journal.pmed.1002672)

**S1 Data Meta-analysis results**

Adding one item at time:

Y=alpha + Beta X

Cesarean event rate= alpha + Beta type of participant (P+ vs. P0)

The model 1 shows that there is a relationship between rate of C/S and type of participant. See inside the blue box.

n:
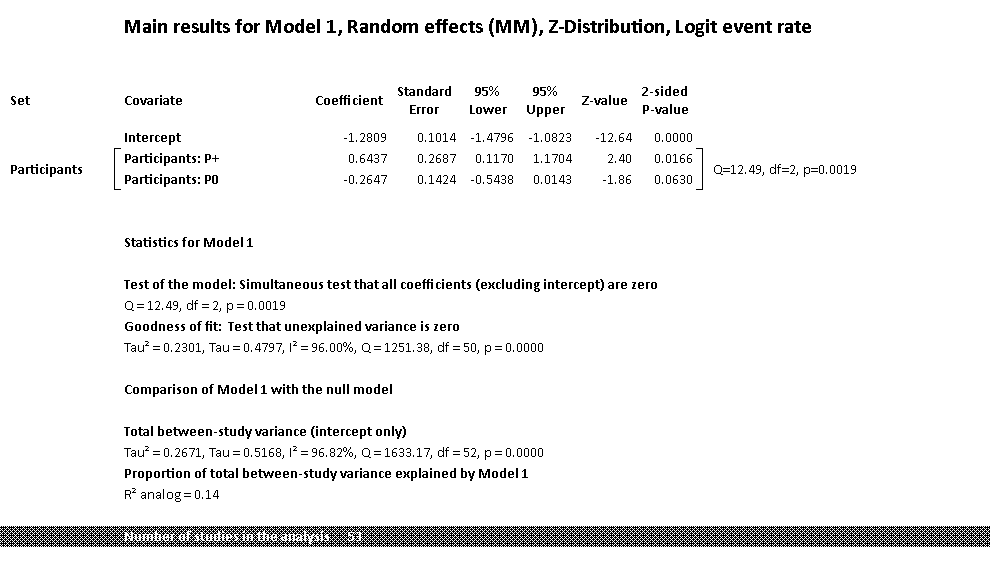


Next, the “study design” was added in the model. This is not significant.


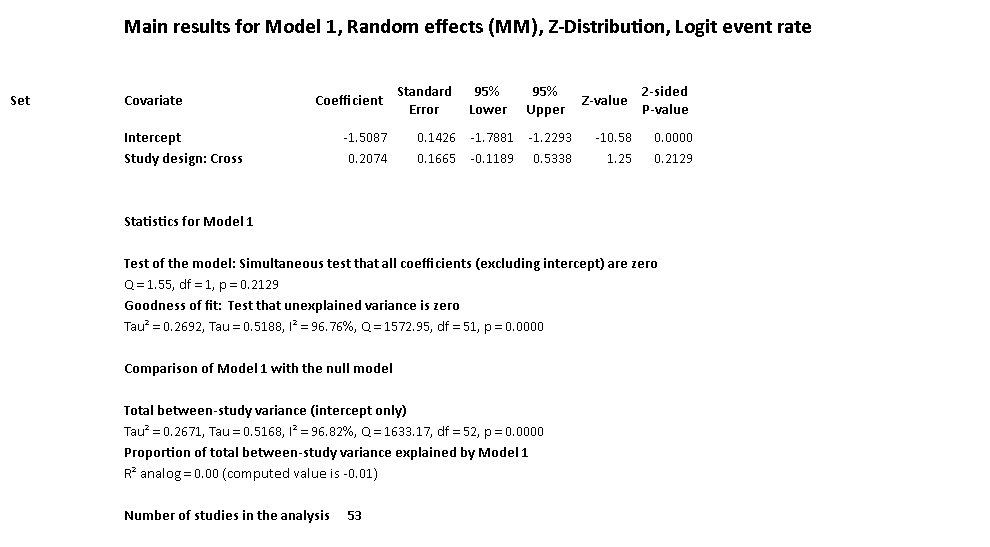


Next, “time point preference reported” was added to the model. This is not significant


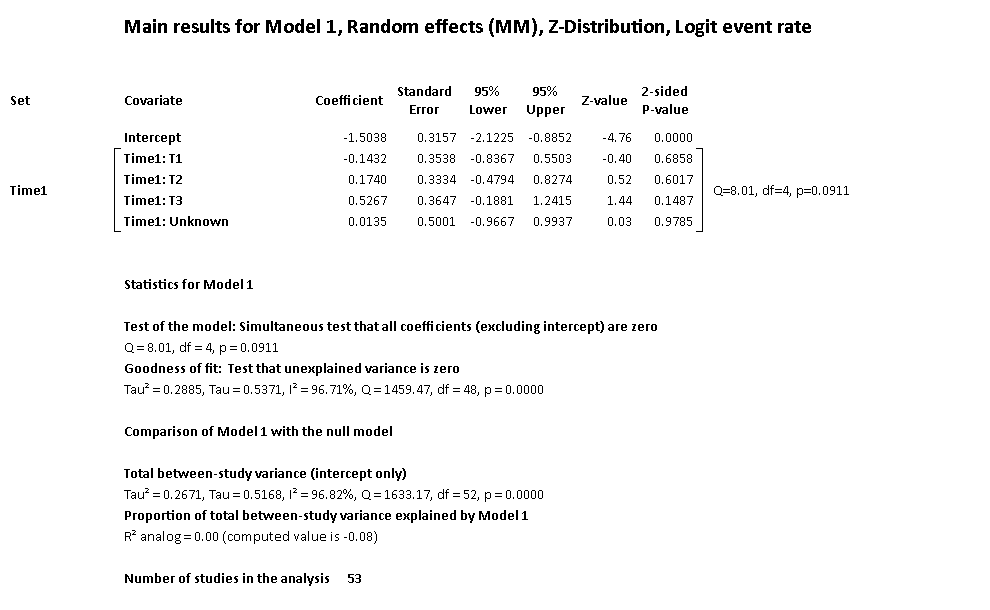


Next, the “location” was added. This is not significant


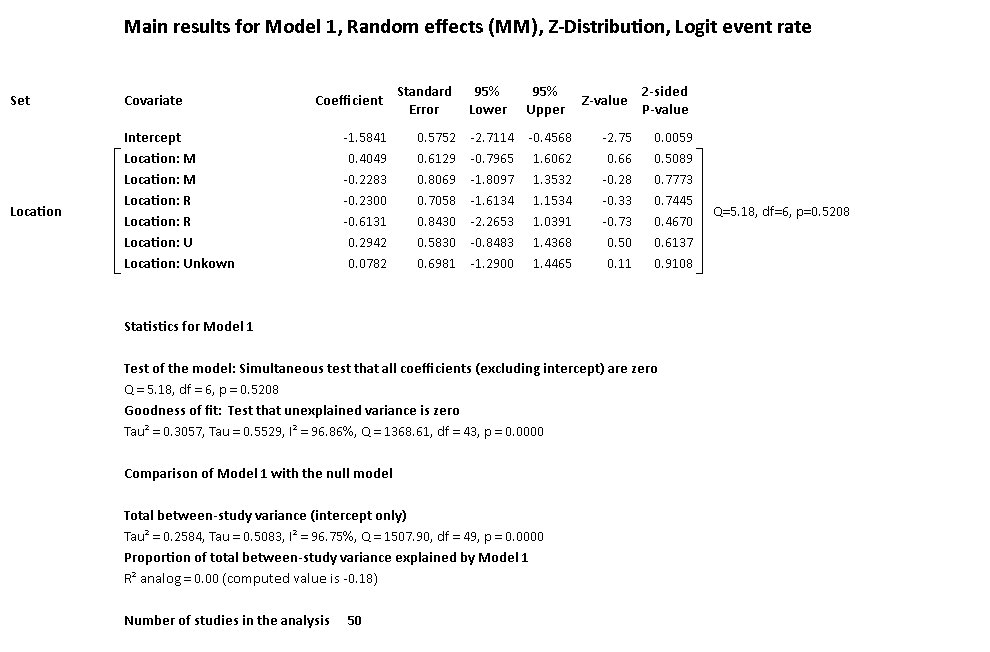


Next, the “region” was added. This is not significant


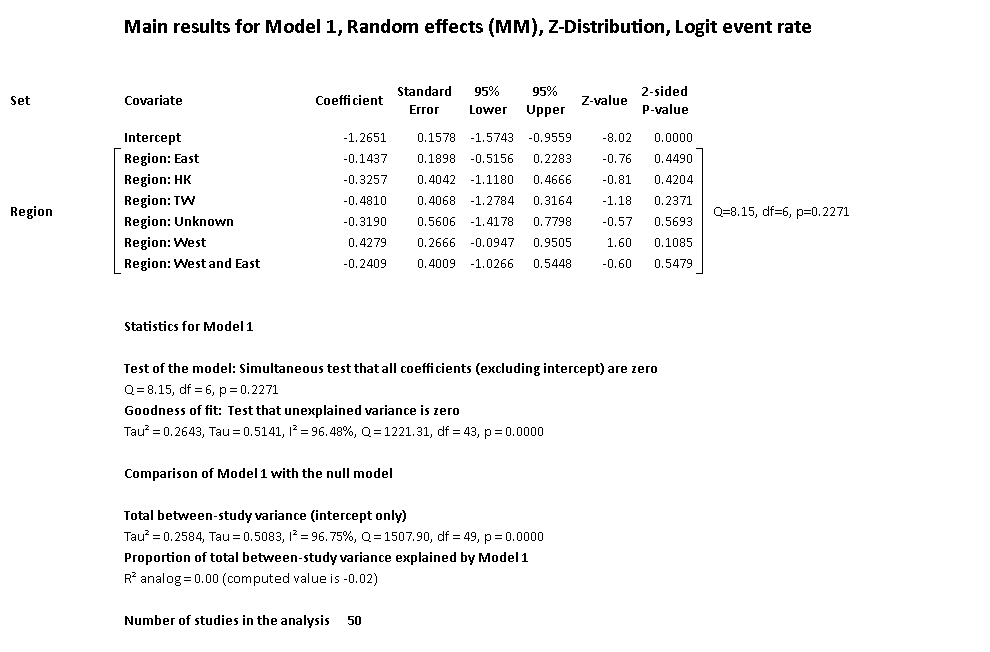


Next, the “risk” of pregnant women (defined by the study authors) was added in the model. This is not significant


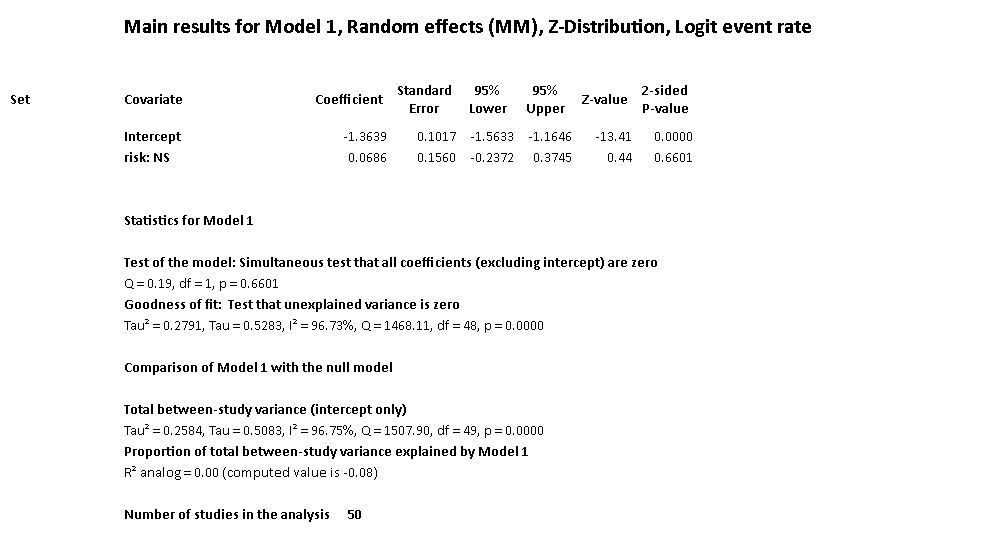


Next, the “quality of studies” (assessed by the review authors) was added. This is not significant


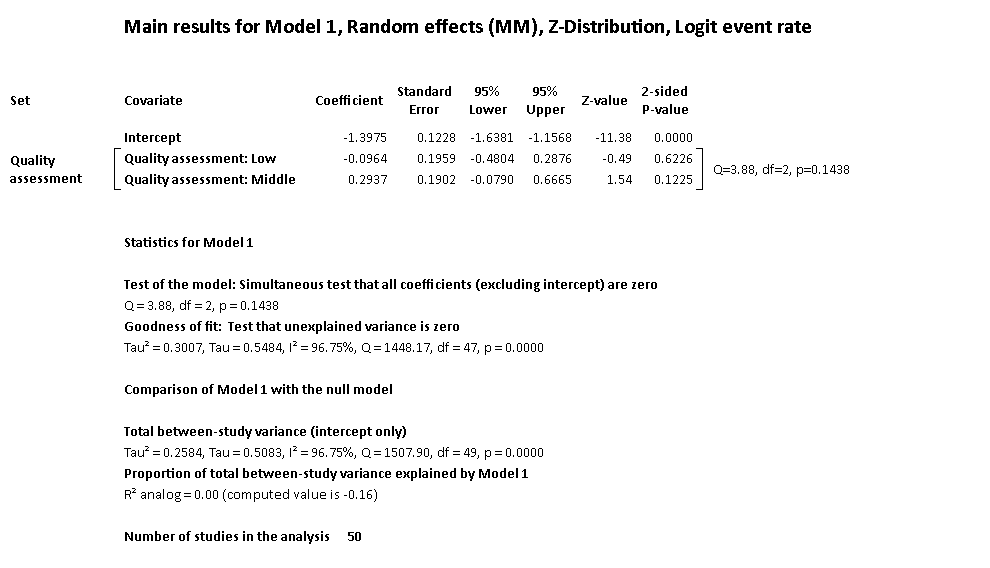


Next, the “level of study facility” was added. This is also not significant


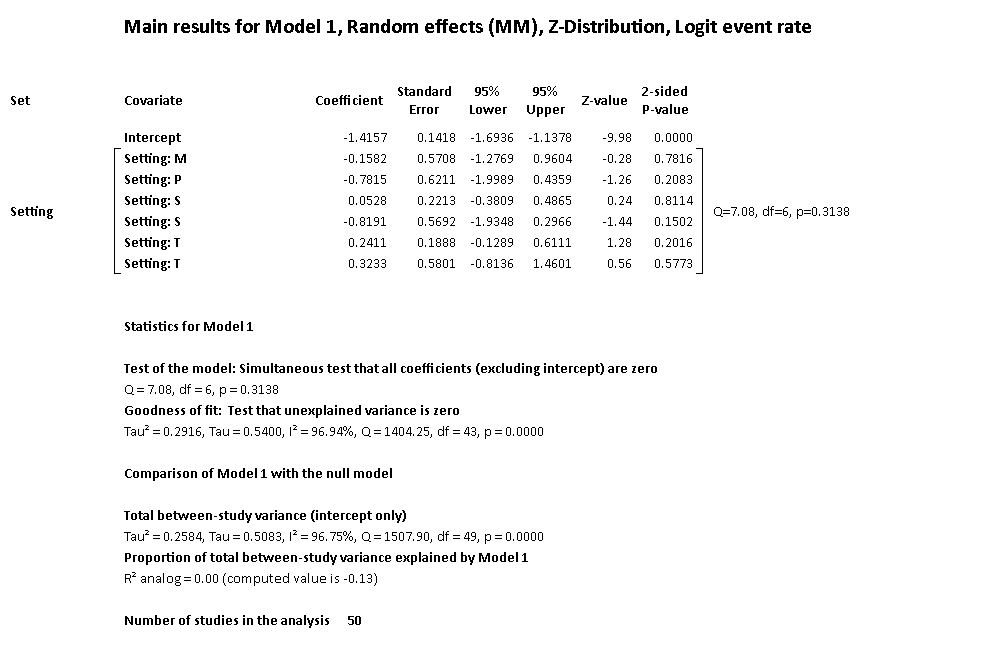


The only variable that has a significant association with event rate is type of participant (nulliparous and multiparous women).

However, the I^2^ in this model was 96%.

Next, a multivariate model was created by adding all the variables in the model irrespective of their significance in univariate model to see how they react in the presence of each other.

There was collinearity issue between location and regions, so location was deleted and the region was kept in the model. The p value is still not significant.

Here is the full model without location


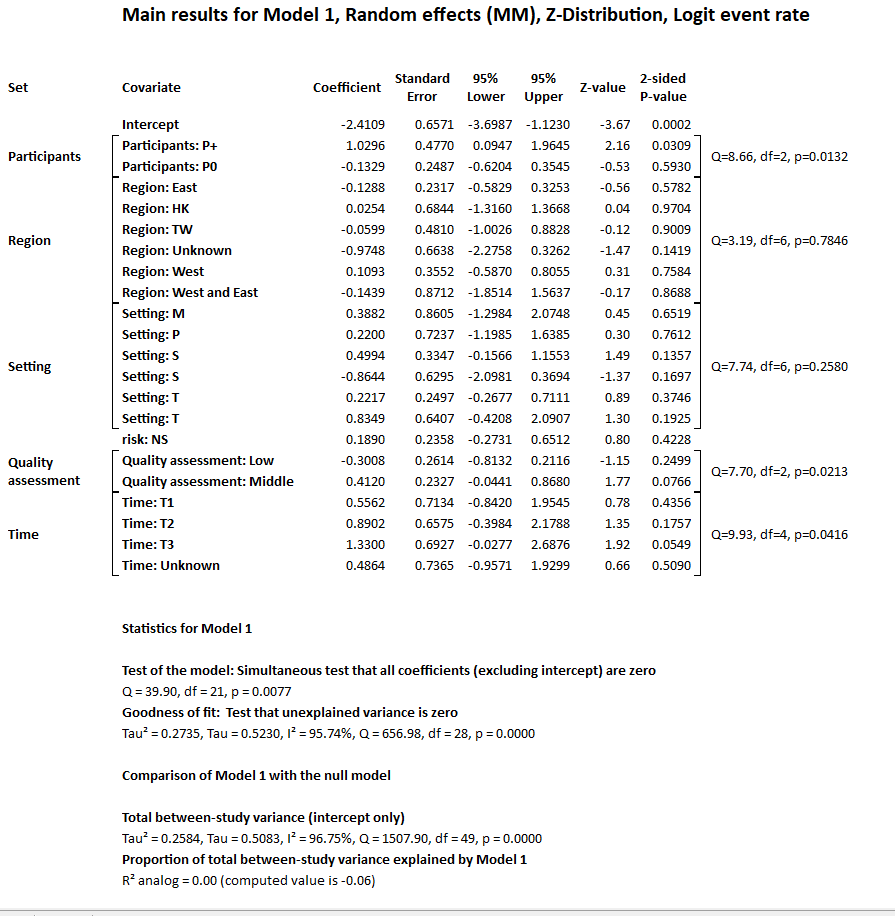


Adding all the items in the model including participant, region, setting, quality assessment and timeline, the model is significant (p=0.0077). The heterogeneity is still high. This means that none of the items used in the model was able to explain the heterogeneity.

Here is the model summary after including all the above-mentioned items in the model:


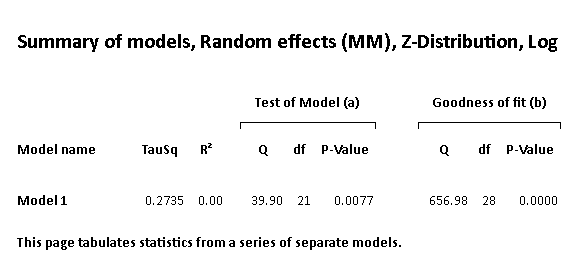


The graph below identifies that the model is not explanatory


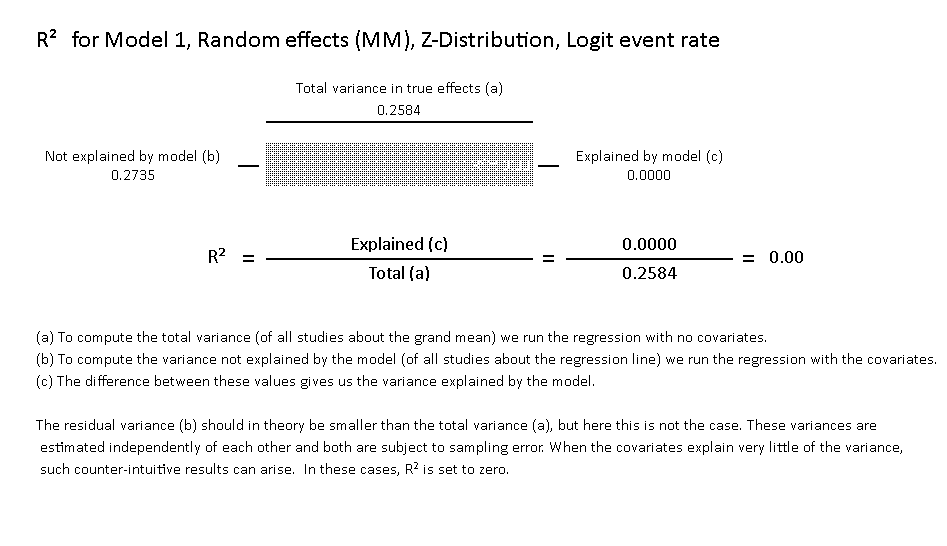


Based on type of participant, this is a graph below:


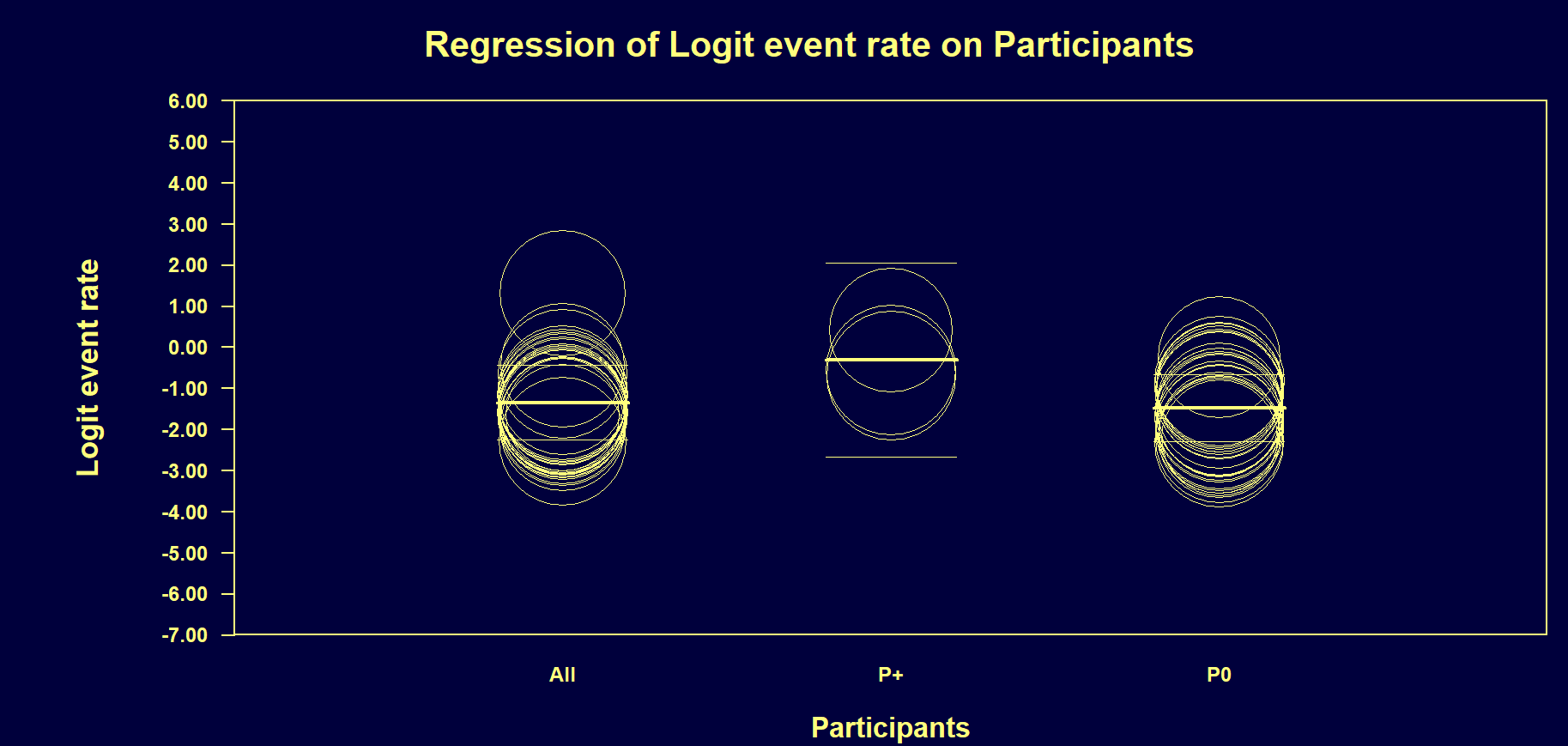


Analysis of publication bias:

Publication bias may be one of the reasons for having such high heterogeneity.


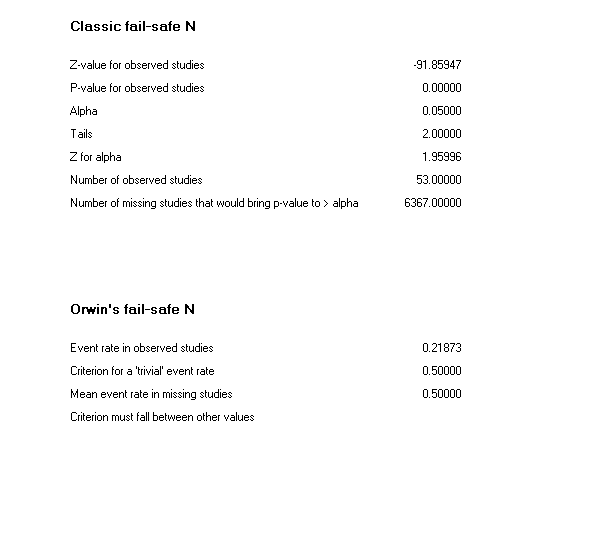

Supplement: S1 Data — (DOCX) [file pmed.1002672.s002.docx]
