# Supplementary material for: Prevalence of and reasons for women’s, family members’, and health professionals’ preferences for cesarean section in China: A mixed-methods systematic review
Source: PLoS Med. 2018 Oct 16;15(10):e1002672. doi: 10.1371/journal.pmed.1002672 (PMC6191094; doi:10.1371/journal.pmed.1002672)
Supplement: S1 Table — (DOCX) [file pmed.1002672.s003.docx]

**S1 Table Search strategies**

*Pubmed / Medline*

*http://www.pubmed.gov*

| # | Searches | Results |
| --- | --- | --- |
| 1 | "Cesarean Section"[Mesh] OR Cesarean [TIAB] OR Caesarean [TIAB] OR “C-sections” [TIAB] OR “C-section”[TIAB] OR “Abdominal Deliveries”[TIAB] OR “Abdominal Delivery”[TIAB] OR Postcesarean [TIAB] OR Postcaesarean [TIAB] OR “Post cesarean” [TIAB] OR “Post caesarean” [TIAB] | 59563 |
| 2 | "Patient Preference"[Mesh] OR "Nurse-Patient Relations"[Mesh] OR "Interviews as Topic"[Mesh] OR "Emotions"[Mesh] OR "Elective Surgical Procedures"[Mesh] OR "Unnecessary Procedures"[Mesh] OR "Physician-Patient Relations"[Mesh] OR "Patient Satisfaction"[Mesh] OR "Natural Childbirth"[Mesh] OR "Health Knowledge, Attitudes, Practice"[Mesh] OR "Choice Behavior"[Mesh] OR "Attitude to Health"[Mesh] OR "Cesarean Section/psychology"[Mesh] OR "Culture"[Mesh] OR "ethnology" [Subheading] OR "Vaginal Birth after Cesarean"[Mesh] OR “decision making” [MH] OR “pain/psychology” [MH] OR “Delivery, obstetric/psychology” [MH] OR “pregnant women/psychology” [MH] OR Patient Participation[Mesh] OR Fear* [TIAB] OR preference* [TIAB] OR prefers [TIAB] OR preferred [TIAB] OR decision* [TIAB] OR “non medical factors” [TIAB] OR “non medical indications” [TIAB] OR option [TIAB] OR options [TIAB] OR optional [TIAB] | 1520832 |
| 3 | Step 1 and Step 2 | 7793 |
| 4 | Publication date from 1990/01/01 to 2016/12/31 | 7191 |

*Note: We set the search date considering the rapid increase of CS rate over the past three decades worldwide. We follow international literature on this topic which usually focus on data from 1990 to assess trends. Recent WHO estimates on trends for caesarean section rates reported data from 1990 as an approximate reasonable date for the unprecedented increase of CS use.*

*PsycINFO*

*http://search.ebsco.com*

| # | Searches | Results |
| --- | --- | --- |
| 1 | DE "Caesarean Birth" OR TI Cesarean OR TI Caesarean OR TI (C-sections) OR TI(C-section) OR TI (Abdominal Deliveries) OR TI (Abdominal Delivery) OR TI Postcesarean OR TI Postcaesarean OR TI (Post cesarean) OR TI (Post caesarean) OR AB Cesarean OR AB Caesarean OR AB (C-sections) OR AB (C-section) OR AB (Abdominal Deliveries) OR AB (Abdominal Delivery) OR AB Postcesarean OR AB Postcaesarean OR AB (Post cesarean) OR AB (Post caesarean) | NA |
| 2 | DE "Preferences" OR DE "Decision Making" OR DE "Emotional States" OR TI Fear* OR TI preference* OR TI prefers OR TI preferred OR TI decision* OR TI (non medical factors)   OR TI (non  medical indications) OR TI option  OR TI options  OR TI optional OR AB Fear* OR AB preference* OR AB prefers OR AB preferred OR AB decision* OR AB (non medical factors)   OR AB (non  medical indications) OR AB option  OR AB options  OR TI optional | NA |
| 3 | Published Date: 1990/01/01-2016/12/31 | 269 |

*CINAHL*

*http://search.ebsco.com*

| # | Searches | Results |
| --- | --- | --- |
| 1 | (MH "Cesarean Section+") OR TI Cesarean OR TI Caesarean OR TI (C-sections) OR TI(C-section) OR TI (Abdominal Deliveries) OR TI (Abdominal Delivery) OR TI Postcesarean OR TI Postcaesarean OR TI (Post cesarean) OR TI (Post caesarean) OR AB Cesarean OR AB Caesarean OR AB (C-sections) OR AB (C-section) OR AB (Abdominal Deliveries) OR AB (Abdominal Delivery) OR AB Postcesarean OR AB Postcaesarean OR AB (Post cesarean) OR AB (Post caesarean) | NA |
| 2 | (MH "Professional-Patient Relations+") OR (MH "Interviews+") OR (MH "Surgery, Elective+") OR (MH "Unnecessary Procedures") OR (MH "Attitude to Health+") OR (MH "Alternative Birth Methods+") OR (MH "Home Childbirth") OR (MH "Prepared Childbirth") OR (MH "Culture+") OR (MH "Ethnological Research") OR (MH "Vaginal Birth+") OR (MH "Health Knowledge") OR (MH "Decision Making, Patient") OR (MH "Decision Support Techniques") OR (MH "Decision Making, Family") OR (MH "Pain/PF") OR (MH "Expectant Mothers/PF") OR (MH "Delivery, Obstetric/PF") OR TI Fear* OR TI preference* OR TI prefers OR TI preferred OR TI decision* OR TI (non medical factors)   OR TI (non  medical indications) OR TI option  OR TI options  OR TI optional OR AB Fear* OR AB preference* OR AB prefers OR AB preferred OR AB decision* OR AB (non medical factors)   OR AB (non  medical indications) OR AB option  OR AB options  OR TI optional | NA |
| 3 | Published Date: 1990/01/01-2016/12/31 | NA |
| 4 | Exclude MEDLINE records | 356 |

*EMBASE*

*http://www.embase.com*

| # | Searches | Results |
| --- | --- | --- |
| 1 | 'cesarean section'/exp OR 'cesarean section' OR 'cesarean section kit'/exp OR 'cesarean section kit' OR cesarean:de,ab,ti OR caesarean:de,ab,ti OR 'c-sections':de,ab,ti OR 'c-section':de,ab,ti OR 'abdominal deliveries':de,ab,ti OR 'abdominal delivery':de,ab,ti OR postcesarean:de,ab,ti OR postcaesarean:de,ab,ti OR 'post cesarean':de,ab,ti OR 'post caesarean':de,ab,ti | 90871 |
| 2 | 'patient preference'/exp OR 'nurse patient relationship'/exp OR 'interview'/exp OR 'emotion'/exp OR 'elective surgery'/exp OR 'unnecessary procedure'/exp OR 'doctor patient relation'/exp OR 'patient satisfaction'/exp OR 'natural childbirth'/exp OR 'attitude to health'/exp OR 'cultural anthropology'/exp OR 'ethnology'/exp OR 'patient participation'/exp OR fear*:de,ab,ti OR preference*:de,ab,ti OR prefers:de,ab,ti OR preferred:de,ab,ti OR decision*:de,ab,ti OR 'non medical factors':de,ab,ti OR 'non medical indications':de,ab,ti OR option:de,ab,ti OR options:de,ab,ti OR optional:de,ab,ti OR (('vaginal birth' OR 'vaginal births') NEAR/2 (cesarean OR caesarean)):de,ab,ti OR ('pregnant woman' NEAR/10 psycholog*):de | 1936849 |
| 3 | Step 1 and Step 2 | 12479 |
| 4 | [1990-2016]/py | 11753 |

*Popline*

[*http://www.popline.org*](http://www.popline.org)

| # | Searches | Results |
| --- | --- | --- |
| 1 | ((Cesarean ) OR (Caesarean ) OR (Cesareans ) OR (Caesareans ) OR (C-sections) OR (C-section) OR (Abdominal Deliveries) OR (Abdominal Delivery) OR (Postcesarean) OR (Postcaesarean)) AND ((Fear) OR (FEARS) OR ( preference) OR (preferences) OR (prefers) OR (prefer) OR (preferred) OR (decision) OR (decisions) OR (non medical factors)   OR (non  medical indications) OR (option)  OR ( options) OR (optional)) | NA |
| 2 | Published Date: 1990-2016 | 414 |

*Global Health Library - Global Index Medicus*

[*http://www.globalhealthlibrary.net/php/index.php*](http://www.globalhealthlibrary.net/php/index.php)

*(Indexes - Regional Indexs) Option selected.*

| # | Searches | Results |
| --- | --- | --- |
| 1 | ((Cesarean ) OR (Caesarean ) OR (Cesareans ) OR (Caesareans ) OR (C-sections) OR (C-section) OR (Abdominal Deliveries) OR (Abdominal Delivery) OR (Postcesarean) OR (Postcaesarean)) AND ((Fear) OR (FEARS) OR ( preference) OR (preferences) OR (prefers) OR (prefer) OR (preferred) OR (decision) OR (decisions) OR (non medical factors)   OR (non  medical indications) OR (option)  OR ( options) OR (optional)) | NA |
| 2 | Published Date: 1990-2016 | 251 |

*One Chinese database_CNKI*

[*http://oversea.cnki.net/kns55/brief/result.aspx?dbPrefix=CJFD*](http://oversea.cnki.net/kns55/brief/result.aspx?dbPrefix=CJFD)

| # | Searches | Results |
| --- | --- | --- |
| 1 | (KY=Preference OR KY=Request OR KY=Demand OR KY=’选择’ OR KY=’要求’ OR KY=’意愿’ OR KY=’决定’ OR TI=Preference OR TI=Request OR TI=Demand OR TI=’选择’ OR TI=’要求’ OR TI=’意愿’ OR TI=’决定’ OR AB=Preference OR AB=Request OR AB=Demand OR AB=’选择’ OR AB=’要求’ OR AB=’意愿’ OR AB=’决定’) AND (KY='剖腹产' OR KY='剖宫产' OR KY=Cesarean OR KY=Caesarean OR TI='剖腹产' OR TI='剖宫产' OR TI=Cesarean OR TI=Caesarean OR AB='剖腹产' OR AB='剖宫产' OR AB=Cesarean OR AB=Caesarean) AND (TI=Study OR TI=’研究’ OR AB=Study OR AB=’研究’) | NA |
| 2 | Select subjects: Medicine & Public Health; Literature/History/Philosophy; Politics/Military Affairs/Law; Education & Social Sciences; Electronic Technology & Information Science; Economics & Management | NA |
| 3 | Published Date: 1990-2016 | 4,480 |

**Search update in 2018**

*English databases 1/1/2017-18/4/2018*

| Database | Results |
| --- | --- |
| Pubmed / Medline | 990 |
| PsycINFO | 63 |
| CINAHL | 175 |
| EMBASE | 3064 |
| Popline | 39 |
| Global Health Library - Global Index Medicus | 55 |
| Total | 4386 |
| Duplicates removed | 3433 |

*One Chinese database _CNKI http://cnki.net/ 1/1/2017-10/5/2018*

| Searches | Results |
| --- | --- |
| SU=('偏好' + '偏爱' + '选择' + '要求' + '意愿' + '决定' + '非医学原因' + '非医疗指针' + '恐惧' + '畏惧')  AND SU=('剖腹产' + '剖宫产'+'分娩') | 200 |
