# Supplementary material for: Prevalence of and reasons for women’s, family members’, and health professionals’ preferences for cesarean section in China: A mixed-methods systematic review
Source: PLoS Med. 2018 Oct 16;15(10):e1002672. doi: 10.1371/journal.pmed.1002672 (PMC6191094; doi:10.1371/journal.pmed.1002672)
Supplement: S3 Table — (DOCX) [file pmed.1002672.s005.docx]

| **S3 Table Assessment of quality of included qualitative studies** | | | | | | | | | | | |
| --- | --- | --- | --- | --- | --- | --- | --- | --- | --- | --- | --- |
| **Author and date** | **Clear aims appropriate to question** | **Literature review thorough and appropriate** | **Theoretical perspective and design clear and appropriate** | **Sampling strategy explained and appropriate** | **Data collection described and justified** | **Analysis adequately described** | **Findings reflect data** | **Study carried out ethically** | **Researcher reflexivity demonstrated** | **Transferability, relevance and usefulness** | **Quality rating ^1^** |
| Lee et al  2001 [26] | Yes | Good - local and international context and debate. | Exploratory, appropriate use of Grounded Theory | Yes - purposive in accordance with methodological approach | Semi-structured interviews | Yes - brief description | Yes - plenty of quotes to support themes | Yes | No | Yes - particularly for midwives | B |
| Wang et al  2006 [78] | Yes | Brief but characteristic of Chinese Journal ^2^ | Qualitative sub-study but no theory | No | Focus group discussions | No - Categorize findings in summary table | Unclear | Unclear | No | No | D |
| Ji  2006 [79] | Yes | Brief but characteristic of Chinese Journal | Applied phenomenological approach (no reference/details/one sentence) | Yes - purposive | Interviews | Yes - brief description | Yes - few quotes to support themes | IRB was not reported but consents of participants were obtained | No | Yes | C- |
| Chen et al  2008 [80] | Yes | Brief but characteristic of Chinese Journal | Semi-structured interviews | Yes | In-depth interviews | Yes - but no reference and steps unfamiliar | Yes - but only one quote for each theme | Yes | No | Yes | D |
| Liu et al  2010 [81] | Yes | Brief but characteristic of Chinese Journal /corresponding thesis chapter | Not reported | Yes pregnant women, family members, HPs | Interviews and focus groups | Yes - Framework analysis, MaxQDA | Yes - few quotes from women but lots from healthcare providers | Yes | No | Yes | C |
| Zhou et al  2012 [82] | Yes | Brief but characteristic of Chinese Journal | Not reported | No | In-depth interviews | Yes - brief description | Yes - few quotes to support themes | Unclear | No | Yes - brief | C |
| Jiang et al  2012 [83] | Yes | Brief but characteristic of Chinese Journal | Mentions phenomenology and Calaizzi as analysis method | Yes -purposive | Interviews | Yes - brief description | Yes - few quotes to support themes | Yes | No | No | C- |
| Zhu et al  2013 [84] | Yes | Brief but characteristic of Chinese Journal | Mentions phenomenology | Yes - purposive | Focus group discussions | Yes - brief description | Yes - many quotes to support themes | Unclear | No | Not really | C |
| Huang et al  2013 [85] | Yes | Good scene setting, no cultural context | Exploratory, appropriate use of Grounded Theory | Yes -followed principles of theoretical sampling | In-depth interviews | Yes- good | Yes - plenty of quotes to support themes | Yes | No | Yes - obstetric focus because of care organization | C |
| Huang  et al 2013  [86] | Yes | Good - international | Limited discussion - sub-study | No | Focus group discussions | Yes - brief description | Yes - not many quotes | Yes | No | Yes | C |
| Wang et al  2013 [87] | Yes | Brief but characteristic of Chinese Journal | Mentions phenomenology and Calaizzi as analysis method | Yes | Semi-structured interviews | Yes - brief description | Yes - few quotes to support themes | Yes | No | Yes - brief in abstract | C |
| Raven et al  2015 [88] | Yes | Good - local and international context and debate. | Yes justified use of qualitative methodology for an exploratory study | Yes -purposive | Semi-structured interviews and focus groups | Yes- good | Yes - plenty of quotes to support themes | Yes | No | Yes | B |
| Chen & Zhang, 2017 [89] | Yes | Brief but characteristic of Chinese Journal | Not reported | No | In-depth interviews, no justification | Yes - brief description | Two quotes in total | Unclear | No | No | D |
| Yang,  2017 [90] | Yes | Good- local and international context and debate/corresponding thesis chapter | Yes justified use of qualitative methodology drawing on phenomenological concepts | Yes- purposive sampling for maximum variation in demographic characteristics | Semi-structured interviews and observation | Yes- good | Yes- many quotes to support themes | Yes | Yes, briefly | May not be transferrable | C+ |
| Wang, 2017 [91] | Yes | Good- local context and debate | Unclear. Use of some terminology suggests Grounded Theory principles | Yes- purposive sampling for variation in parity and birth outcomes | Semi-structured interviews and observations | Yes- brief description | Yes - many quotes to support themes | Yes | No | May not be transferrable, particularly in less developed areas | B+ |
| Wang & Hesketh, 2017 [92] | Yes | Good - local and international context and debate. | Semi-structured interviews and observations | Yes-convenience sampling | Semi-structured interviews and observations | Yes-brief description | Yes -- many quotes to support themes | Yes | Yes | May not be transferrable | A- |
| Chen et al  2018 [93] | Yes | Good- local and international context and debate | Yes justified use of grounded theory with adherence to principles evident throughout paper | Yes – purposive sampling briefly mentioned without details | Non-participant observation, in-depth interviews and field notes | Yes – good | Yes- plenty of quotes to support themes | Yes | Yes | May not be transferrable to all pregnant women in Taiwan, but still relevant due to its depth | A- |
| Gu et al  2018 [94] | Yes | Good - local and international context and debate. | Yes justified use of qualitative methodology for an exploratory study | Yes- purposive sampling for maximum variation in demographic characteristics | Semi-structured interviews | Yes- adequate description in accordance with the methodology | Yes - plenty of quotes to support themes | Yes | No | Yes - particularly for the post-policy change period of time and similar clinical context | B |
| Chen et al  2017 [95] | Yes | Good- local and international context and debate | Yes justified use of qualitative methodology guided by Theory of Planned Behavior | No | Interviews | Yes- adequate description in accordance with the methodology | Yes- plenty of quotes to support themes | Yes | No | Yes- can be used for the basis for a large study | B |

*^1^Key to quality rating:-* **A** No or few flaws. The study credibility, transferability, dependability, and confirmability is high; **B** – Some flaws, unlikely to affect the credibility, transferability, dependability, and/or confirmability of the study; **C** – Some flaws which may affect the credibility, transferability, dependability, and/or confirmability of the study; **D** – Significant flaws which are very likely to affect the credibility, transferability, dependability, and/or confirmability of the study.

^2^ The length of a Chinese academic article is often 4-5 pages, and the introduction of the study is often short.
