# Supplementary material for: Prevalence of and reasons for women’s, family members’, and health professionals’ preferences for cesarean section in China: A mixed-methods systematic review
Source: PLoS Med. 2018 Oct 16;15(10):e1002672. doi: 10.1371/journal.pmed.1002672 (PMC6191094; doi:10.1371/journal.pmed.1002672)
Supplement: S4 Table — (DOCX) [file pmed.1002672.s006.docx]

**S4 Table Characteristics of the included studies**

| **Study** | **Language of publication** | **Year of data collection** | **Region** | **Location** | **Population vs. facility based** | **No. of study sites** | **Participants**  **(n)** | **Study design** | **Method** | **Outcomes** | **Time of preference reported** | **Quality assessment** |
| --- | --- | --- | --- | --- | --- | --- | --- | --- | --- | --- | --- | --- |
| **Quantitative studies** | | | | | | | | | | | | |
| Cao et al., 2004 [37] | Chinese | 2001-2002 | Shanghai, East | Urban | Facility-based | Not reported | Pregnant women (n=565, parity unknown) | Cross-sectional | Face-to-face interview | Preference for a MOD for current pregnancy and reasons | Early pregnancy | Low |
| Ma et al., 2004 [45] | Chinese | 2002-2003 | Xinjiang, West | Urban | Facility-based | 1 (secondary) | Nulliparous pregnant women (n=787) | Cross-sectional | Face-to-face interview | Preference for a MOD for current pregnancy | Late pregnancy | Low |
| Fan et al., 2005 [74] | Chinese | 2003 | Shanghai, East | Urban | Facility-based | 18 (mixed) | Maternity care providers (n=462) | Cross-sectional | Self-administered | 1) Preference for CS in general  2) Perceptions of a MOD | N/A | Low |
| Wang et al., 2005# [47] | Chinese | 2001-2002 | Shanghai, East | Urban | Facility-based | 3 (secondary) | Nulliparous pregnant women without serious diseases (n=931) | Cross-sectional (Longitudinal design) | Face-to-face interview | Preference for a MOD for current pregnancy | Late pregnancy | High |
| Pang et al., 2007* [8] | English | 2003-2004 | Hong Kong | Urban | Facility-based | 2 (mixed) | Nulliparous pregnant women who had singleton and were eligible for a trial of VD (n=501) | Cross-sectional (Longitudinal design) | Face-to-face interview | Preference for a MOD for current pregnancy and reasons | Early or middle | High |
| Pang et al., 2007 [39] | English | 2002 | Hong Kong | Urban | Facility-based | 1 (tertiary) | Nulliparous and Multiparous pregnant women who were eligible for a trial of VD (n=629;p0=370) | Cross-sectional | Face-to-face interview | Preference for a MOD for current pregnancy and reasons | Early pregnancy | High |
| Wang et al., 2007 [52] | Chinese | 2003 | Beijing, East | Urban and peri-urban | Facility-based | 9 (mixed) | Postpartum women (n=826, parity unknown) | Cross-sectional | Face-to-face interview | 1) Preference for a MOD for index birth  2) Perceptions of a MOD | Postpartum (7 days after the birth) | Low |
| Song et al., 2007 [46] | Chinese | 2006 | Beijing, East | Urban | Facility-based | 1 (secondary) | Nulliparous pregnant women without serious diseases and pregnancy related complications (n=216) | Cross-sectional | Self-administered | Preference for a MOD for current pregnancy and reasons for the preference | Late pregnancy | Low |
| Chu et al., 2010 [9] | English | 2006-2007 | Taiwan | Urban | Facility-based | 4 (mixed) | Pregnant women who had no pre-existing conditions or complications during early pregnancy (n=473;p0=281) | Longitudinal | Mail survey | Preference for a MOD for current pregnancy | Early or middle and late pregnancy | High |
| Han et al., 2010 [35] | Chinese | 2009 | Shanghai, East | Urban and peri-urban | Facility-based | 38 (mixed) | Maternity care providers (n=342) and postpartum women (n=570, parity unknown) | Longitudinal | Face-to-face interview | 1) Preference for CS for index birth 2) Maternity care providers’ perceptions on CS | Postpartum to recall preference in early and late pregnancy | High |
| Jiang & Li, 2012 [44] | Chinese | 2010-2011 | Zhejiang, East | Urban | Facility-based | 1 (secondary) | Nulliparous pregnant women without pregnancy related complications and other diseases (n=253) | Cross-sectional | Face-to-face interview | Preference for a MOD for current pregnancy and reasons | Late pregnancy | Low |
| Xue & Zhang, 2012 [49] | Chinese | Unknown | Jilin, East North | Urban | Facility-based | 1 (secondary) | Nulliparous pregnant women without severe diseases (n=100) | Cross-sectional | Self-administered | Preference for a MOD for current pregnancy | Late pregnancy | Middle |
| Zhang et al., 2012 [54] | Chinese | 2010-2011 | Hebei, East | Urban | Facility-based | 3 (secondary) | Pregnant women (n=400, parity unknown) | Cross-sectional | Self-administered | Preference for a MOD for current pregnancy and reasons | During pregnancy (gestational age not reported) | Low |
| Zhou et al., 2012 [53] | Chinese | 2011 | Shanghai, East | Urban | Facility-based | 1 (tertiary) | Low risk of nulliparous postpartum women (n=336) | Cross-sectional | Face-to-face interview | Preference for a MOD for index birth | Postpartum (before discharge) | High |
| Sun et al., 2013 [41] | Chinese | 2011-2012 | Hubei, Central | Urban | Facility-based | 1 (tertiary) | Nulliparous pregnant women (n=519) | Experiment (baseline) | Questionnaire survey | Preference for a MOD for current pregnancy | Early or middle pregnancy | Low |
| Deng et al., 2014 [15] | English | 2006-2007 | Shanghai, East | Urban | Facility-based | 1 (tertiary) | Nulliparous postpartum women (n=263) | Longitudinal | Self-administered | Preference for a MOD for index birth | Postpartum to recall preferences in early and late pregnancy | High |
| Gao, 2014 [42] | Chinese | 2012-2013 | Shanxi, Central | Rural | Facility-based | 1 (secondary) | Nulliparous pregnant women without severe diseases, pregnancy related complications and medical indications for CS (n=485) | Cross-sectional | Self-administered | Preference for a MOD for current pregnancy | Late pregnancy | High |
| Li et al., 2014 [36] | English | 2011 | Beijing, East | Urban | Facility-based | 1 (tertiary) | Nulliparous pregnant women without serious medical or psychiatric diseases; and Women’s partner (n=257) | Longitudinal | Face-to-face interview | Preference for a MOD for current pregnancy and index birth (if they could choose again) and reasons | Late pregnancy and 2 or 3 days after birth | High |
| Li et al., 2014 [51] | Chinese | 2006-2011 | Shanxi, Central | Urban | Facility-based | 1 (tertiary) | Postpartum women (n=2778, parity unknown) | Cross-sectional | Self-administered | Preference for a MOD for index birth | Postpartum (six weeks after the birth) | Middle |
| Liang et al., 2014 [38] | Chinese | 2011 | Shanghai, East | Urban | Facility-based | 1 (secondary) | Pregnant women (n=325, parity unknown) | Experiment (baseline) | Face-to-face interview | Preference for CS for current pregnancy | Early pregnancy (before the intervention) | Low |
| Xin, 2014 [48] | Chinese | 2012-2013 | Fujian, East | Urban | Facility-based | 1 (tertiary) | Pregnant women (n=80, parity unknown) | Cross-sectional | Self-administered | 1) Preference for a MOD for current pregnancy  2) Perceptions of a MOD | Late pregnancy | Low |
| Loke et al., 2015 [73] | English | 2013 | Hong Kong | Urban | Facility-based | Unknown | Women of childbearing age (pregnant or had given birth within past three years) | Cross-sectional | Self-administered | 1) Preference for a MOD in general and reasons  2) Perceptions of CS | N/A | High |
| Song, 2015 [40] | Chinese | 2013-2014 | Jiangsu, East | Rural | Facility-based | 1 (primary) | Nulliparous pregnant women without pregnancy related complications (n=150) | Experiment (baseline) | Not reported | Preference for a MOD for current pregnancy | Early pregnancy (before the intervention) | Low |
| Ji et al., 2015 [14] | English | 2010-2011 | Shanghai, East | Urban | Facility-based | 2 (mixed) | Nulliparous pregnant women without severe diseases and mental illness (n=832) | Longitudinal | Self-administered | Preference for a MOD for current pregnancy | Early or middle and late pregnancy | High |
| Gong et al., 2016 [43] | Chinese | 2010 | Unknown | Unknown | Facility-based | 1 (secondary) | Pregnant women without severe diseases or pregnancy-related complications (n=282, parity unknown) | Cross-sectional | Face-to-face interview | 1) Preference for a MOD for current pregnancy  2) Perceptions of a MOD | Late pregnancy | High |
| Zhang et al., 2016 [50] | Chinese | 2013 | Jiangxi, Central | Mixed (urban and rural) | Facility-based | 13 (mixed) | Pregnant women without serious diseases and pregnancy related complications (n=12143, parity unknown); and Their family members (n=12143) | Cross-sectional | Face-to-face interview | Preference for a MOD for current pregnancy and reasons | Late pregnancy | High |
| Gao et al., 2017 [55] | Chinese | 2012 | Hunan, Central | Mixed (urban and rural) | Facility-based | 6 (mixed) | Women giving birth within a year and no pregnancy related complications during the birth (n=603, parity unknown) | Experiment (baseline) | Self-administered | Preference for a MOD for index birth and reasons | Postpartum (birth within a year) | High |
| Lei et al., 2017 [56] | Chinese | 2013 | Shanghai, East | Urban | Facility-based | 8 (unknown) | Nulliparous and multiparous pregnant women without pregnancy related risks or complications (n=1559; p0=1299) | Cross-sectional | Face-to-face interview | Preference for a MOD for current pregnancy | Late pregnancy | Middle |
| Li, 2017 [57] | Chinese | 2013 | Jiangsu, East | Urban | Facility-based | 1 (secondary) | Nulliparous and multiparous pregnant women without pregnancy related complications (n=140) | Cross-sectional | Face-to-face interview | Preference for a MOD for current pregnancy | Late pregnancy | Middle |
| Zheng et al., 2017 [58] | Chinese | 2013 | Hunan, Central | Urban | Facility-based | 4 (mixed) | Postpartum women (n=952, parity unknown) | Cross-sectional | Face-to-face interview | Preference for CS for index birth | Postpartum | Low |
| Lin & Zhang, 2017 [59] | Chinese | 2012-2013 | Jilin, East | Urban | Facility-based | 1 (tertiary) | Postpartum women (n=264, parity unknown) | Cross-sectional | Face-to-face interview | Preference for a MOD for index birth | Postpartum | Middle |
| Wang, 2017 ^ [60] | Chinese | 2014 | Tianjin, East | Urban | Facility-based | 1 (tertiary) | Postpartum women (n=582; P0=481) | Cross-sectional | Self-administered | Preference for a MOD and reasons of preferring for CS | Postpartum | Low |
| Huang et al., 2017 [61] | Chinese | 2015 | Anhui, Central | Mixed (urban and rural) | Facility-based | 8 (mixed) | Nulliparous and multiparous pregnant women at early, middle and late pregnancy (n=1159) and family member (n=408) | Cross-sectional | Face-to-face interview or Self-administered | Preference for a MOD for current pregnancy | Prenatal check-up | Middle |
| Xu & Deng, 2017 [62] | Chinese | 2016 | Hubei, Central | Urban | Facility-based | 1 (tertiary) | Nulliparous pregnant women without CS indications and other diseases (n=449) | Cross-sectional | Face-to-face interview | Preference for a MOD for current pregnancy and reasons | Late pregnancy | Low |
| Xie & Tan, 2017 [63] | Chinese | 2016 | Guangdong, East | Urban | Facility-based | 3 (tertiary) | Nulliparous and multiparous pregnant women without CS indications and other diseases (n=244) | Cross-sectional | Self-administered | Preference for a MOD for current pregnancy and reasons | Late pregnancy | High |
| Liao et al., 2017 [64] | Chinese | 2016 | Hunan and Hubei, Central | Urban | Facility-based | 2 (secondary) | Nulliparous and multiparous pregnant women without CS indications and other diseases (n=400) | Cross-sectional | Face-to-face interview | Preference for a MOD for current pregnancy | Prenatal check-up | Middle |
| Zhang & Xiang, 2017 [65] | Chinese | 2015 -2016 | Zhejiang, East | Rural | Facility-based | 1 (secondary) | Nulliparous pregnant women without CS indications and other diseases (n=188) | Cross-sectional | Face-to-face interview | Preference for a MOD for current pregnancy | Late pregnancy | High |
| Xie & Guo, 2017 [66] | Chinese | 2016-2017 | Xinjiang, West | Urban | Facility-based | 2 (tertiary) | Multiparous pregnant women without pregnancy related complications (n=1237) | Cross-sectional | Face-to-face interview | Preference for a MOD for current pregnancy | Late pregnancy | High |
| Zhang et al., 2017 [67] | Chinese | 2014-2016 | Sichuan, West | Urban | Facility-based | 1 (tertiary) | Nulliparous and multiparous pregnant women aged at 35-43 without pregnancy related complications (n=384) | Cross-sectional | Face-to-face interview | Preference for a MOD for current pregnancy | Late pregnancy | Middle |
| Zhao et al, 2017 [68] | English | 2016 | Chongqing, West | Urban | Facility-based | 1 (tertiary) | Nulliparous pregnant women without pregnancy related complications (n=814) | Longitudinal | Self-administered | Preference for a CS for current pregnancy | Middle and late pregnancy | High |
| Zhang et al, 2018 [69] | English | 2015 | Shanghai, East | Urban | Facility-based | 1 (tertiary) | Nulliparous pregnant women (n=1211) | Longitudinal | Self-administered | Preference for a MOD for current pregnancy | Middle and late pregnancy | High |
| Zhang et al, 2017 [70] | English | 2014 | Beijing, East | Urban | Facility-based | 1 (tertiary) | Nulliparous and multiparous pregnant women (n=450) | Cross-sectional | Self-administered | Preference for a MOD for current pregnancy and reasons | Late pregnancy | High |
| Shi et al, 2016 [71] | English | 2012 | Hunan, Central | Mixed (urban and rural) | Facility-based | 6 (mixed) | Nulliparous and multiparous pregnant women without pregnancy related complications (n=375) and women giving birth within a year (n=602) | Cross-sectional | Self-administered | Preference for a MOD for current pregnancy or index birth | Late pregnancy or postpartum | High |
| Wang et al, 2016 [72] | English | 2015 | Chongqing, Sichuan and Guizhou in West and Tianjing and Shandong in East | Unknown | Facility-based | 16 (mixed) | Nulliparous and multiparous pregnant women (n=2345) | Cross-sectional | Face-to- face interview | Preference for a MOD for current pregnancy | Prenatal periods | Middle |
| Chen et al, 2017 [75] | Chinese | 2015-2016 | Guangdong, East | Urban | Facility-based | 1 (tertiary) | Pregnant women with previous CS (n=388) | Longitudinal | Face-to- face interview in the first survey and telephone survey in the follow-up | Preference for a MOD for current pregnancy | Early or middle and late pregnancy | High |
| Wu et al, 2017 [76] | Chinese | 2014-2017 | Guangdong, East | Urban | Facility-based | 1 (secondary) | Pregnant women with previous CS (n=240) | Cross-sectional | Face-to- face interview | Preference for a MOD for current pregnancy and reasons | Prenatal check-up | Middle |
| Yan et al, 2018 [77] | Chinese | 2015 | Shanxi, Central | Urban | Facility-based | 1 (tertiary) | Pregnant women with previous CS (n=318) | Cross-sectional | Self-administered | Preference for a MOD for current pregnancy and reasons | Prenatal check-up | Middle |
| **Qualitative studies** | | | | | | | | | | | | |
| Lee et al., 2001 [26] | English | Unknown | Hong Kong | Urban | Facility-based | 1 (private hospital) | Nulliparous women who requested CS (n=6) | Qualitative | IDIs | Reasons for choosing an elective CS | Postpartum | B |
| Wang et al., 2006 # [78] | Chinese | 2001-2002 | Shanghai, East | Urban | Facility-based | 3 (secondary) | Nulliparous postpartum women having CS (n=12) and having VD (n=15) | Qualitative | FGDs | Perceptions of women on a MOD | Postpartum (three month after birth) | D |
| Ji, 2006 [79] | Chinese | Unknown | Beijing, East | Urban | Facility-based | 2 (tertiary) | Nulliparous pregnant women who wish to have CS (n=10) | Qualitative | IDIs | Reasons of wishing to have CS | Late pregnancy | C- |
| Chen et al., 2008 [80] | Chinese | Unknown | Shanghai, East; Guangxi (Liuzhou and Yizhou), West | Urban | Mixed | 5 hospitals and some other contacts | Women having CS (n=51) and healthcare professionals (n=51) | Qualitative | IDIs | Impact of informed consent on choosing CS | Postpartum (one week to eight months after birth) | D |
| Liu et al., 2010 [81] | Chinese | 2008 | Anhui, Central | Rural | Mixed | 2 counties | Pregnant women (n=19), women having VD (n=16) and having CS (n=18) and family members (n=20); Health professionals (n=9) | Qualitative | FGDs and IDIs | Perceptions of stakeholders on CS | Late pregnancy and postpartum | C |
| Zhou et al., 2012 [82] | Chinese | 2012 | Shanghai, East | Urban | Facility-based | 1 (tertiary) | Nulliparous women who had NMCS (n=25) | Qualitative | IDIs | Reasons for choosing NMCS | Postpartum | C |
| Jiang et al., 2012 [83] | Chinese | 2011 | Guangxi, West | Urban | Facility-based | 1 (tertiary) | Nulliparous women who had NMCS (n=10) | Qualitative | IDIs | Reasons for choosing NMCS | Postpartum | C- |
| Zhu et al., 2013 [84] | Chinese | Unknown | Hunan, Central | Urban | Facility-based | 6 (mixed) | Policy makers and health managers (n=10) | Qualitative | FGDs | Current hinders of VD | N/A | C |
| Huang et al., 2013 [85] | English | 2009-2010 | Taiwan | Unknown | Mixed | N/A | Nulliparous women (n=20) | Qualitative | IDIs | Women’s decision making process | Postpartum | C |
| Huang et al., 2013 [86] | English | 2005-2006 | Anhui, Central | Rural | Population-based | 2 counties | Postpartum women, township doctors and village family planning workers (total n=58) | Qualitative | FGDs | Reasons for choosing CS | N/A | C |
| Wang & Ding 2013 [87] | Chinese | Unknown | Shanghai, East | Urban | Facility-based | 1 (unknown) | Nulliparous maternity care providers who had NMCS (n=11) | Qualitative | IDIs | Reasons for choosing NMCS | Postpartum | C |
| Raven et al., 2015 [88] | English | 2007-2009 | Anhui, Central | Rural | Facility-based | 8 (mixed) | Postpartum women (nulliparous and multiparous, n=69) | Qualitative | IDIs and FGDs | Women’s perceptions and experience of the quality of care | Postpartum | B |
| Chen & Zhang, 2017 [89] | Chinese | 2016-2017 | Shandong, East | Urban | Facility-based | 1 (secondary) | Pregnant women who had NMCS (n=8) | Qualitative | IDIs | Reasons for choosing NMCS | Unclear | D |
| Yang, 2017 [90] | Chinese | 2016 | Zhejiang, East | Urban | Facility-based | 1 (maternity hospital) | Pregnant women with previous CS (n=21), Doctors (n=7) and Midwives (n=8) | Qualitative | IDIs | Experience and needs of women in the process of choosing vaginal birth after CS | Antenatal care | C+ |
| Wang, 2017 [91] | English | 2015 | Shanghai, East | Urban | Facility-based | 1 (secondary) | Postpartum women (nulliparous and multiparous, n=26) and maternity care providers (n=8) | Qualitative | IDIs | Perceptions of labor pain related to request for CS | Postpartum | B+ |
| Wang & Hesketh, 2017 [92] | English | 2016-2017 | Zhejiang, East | Mixed (urban and rural) | Facility-based | 2 (tertiary and secondary) | Postpartum women (nulliparous and multiparous, n=45); Healthcare providers (n=7) | Qualitative | IDIs | Stakeholders’ attitude of childbirth and experience of delivery decision-making | Postpartum | A- |
| Chen et al., 2018 [93] | English | Unknown | Taiwan | Unknown | Facility-based | 1 (tertiary) | Pregnant women with previous CS (n=21) and obstetricians (n=9) | Qualitative | IDIs | Women’s decision-making processes on a MOD following a previous CS | Antenatal and postnatal visits | A- |
| Gu et al., 2018 [94] | English | 2015-2016 | Shanghai, East | Urban | Facility-based | 1 (tertiary) | Nulliparous women (n=21) | Qualitative | IDIs | Women’s decision making on a MOD | Postpartum | B |
| Chen et al., 2017 [95] | English | 2010 | Taiwan | Unknown | Facility-based | 2 (mixed) | Pregnant women with previous CS (n=29) and postpartum women (n=35) | Qualitative | IDIs | Women’s decision making on a MOD following a previous CS | Prenatal and postnatal | B |

* The study Pang 2007a was a longitudinal design. Twenty-two women with “absolute indications for caesarean section” were excluded at 37 weeks follow-up. This study was considered as a cross-sectional study and the reported preference for CS in the initiation of the study was included in the analysis.

# The study Wang 2005/Wang 2006 was a longitudinal design. In Wang 2006, the postpartum women who had caesarean section with medical indications were excluded. The study Wang 2005 was considered as a cross-sectional study. The study Wang 2006 was a mixed methods study and qualitative component was included in the review.

^ The study Wang 2017 was a mixed method study. The study did not report analysis method for qualitative component, and thus only quantitative component was included.

CS: Caesarean section; FGD: Focus group discussion; IDI: In-depth interview; MOD: mode of delivery; NMCS: Non-medically indicated caesarean section; VD: vaginal delivery
