# Supplementary material for: Prevalence of and reasons for women’s, family members’, and health professionals’ preferences for cesarean section in China: A mixed-methods systematic review
Source: PLoS Med. 2018 Oct 16;15(10):e1002672. doi: 10.1371/journal.pmed.1002672 (PMC6191094; doi:10.1371/journal.pmed.1002672)
Supplement: S5 Table — (DOCX) [file pmed.1002672.s007.docx]

**S5 Table Uncertain preference for a mode of delivery for current pregnancy, quantitative surveys**

| **Studies** | **Region** | **Year of data collection** | **Participants** | **Number of participants** |  | **Early or middle pregnancy** | | **Late pregnancy** | |
| --- | --- | --- | --- | --- | --- | --- | --- | --- | --- |
|  |  |  |  |  |  | **n** | **%** | **n** | **%** |
| *Longitudinal studies* | | | | | | | | | |
| Chu et al., 2010 [9] | Taiwan | 2006-2007 | Nulliparous and multiparous women | T1: 473  T2: 473 | Undecided | 76 | 16.1 | 19 | 4.0 |
| Deng et al., 2014 [15] | Shanghai, East | 2006-2007 | Nulliparous women | T1: 215  T2: 215 | No preference | 27 | 12.6 | 23 | 10.7 |
| Ji et al., 2015 [14] | Shanghai, East | 2010-2011 | Nulliparous women | T1: 832  T2: 599 | No preference | 122 | 14.7 | 16 | 2.7 |
| Zhang et al., 2018 [69] | Shanghai, East | 2015 | Nulliparous women | T1: 1211  T2: 1147 | Not considered yet | 126 | 10.4 | 67 | 5.8 |
| *Cross-sectional studies* | | | | | | | | | |
| Ma & Zhang, 2004 [45] | Xinjiang, West | 2002-2003 | Nulliparous women | 787 | Adhere to arrangement | -- | -- | 270 | 34.3 |
| Wang et al., 2005 [47] | Shanghai, East | 2001-2002 | Nulliparous women | 931 | Fine with both MOD | -- | -- | 111 | 11.9 |
| Song et al., 2007 [46] | Beijing, East | 2006 | Nulliparous women | 216 | Unable to decide | -- | -- | 45 | 20.8 |
| Zhang et al., 2012* [54] | Hebei, East | 2010-2011 | Pregnant women (parity unknown) | 400 | Fine with both MOD | -- | -- | 85 | 21.2 |
| Jiang & Li, 2012 [44] | Zhejiang, East | 2010-2011 | Nulliparous women | 253 | Fine with both MOD | -- | -- | 70 | 27.7 |
| Xue & Zhang, 2012 [49] | Jilin, East North | Unknown | Nulliparous women | 100 | Fine with both MOD | -- | -- | 29 | 29.0 |
| Xin, 2014 [48] | Fujian, East | 2012-2013 | Pregnant women (parity unknown) | 80 | Not consider it | -- | -- | 5 | 6.3 |
| Gong et al., 2016 [43] | Unknown | 2010 | Pregnant women (parity unknown) | 282 | No preference | -- | -- | 27 | 9.6 |
| Wang, 2016 [72] | Chongqing, Sichuan and Guizhou in West and Tianjin and Shandong in East | 2015 | Nulliparous and multiparous women | T1: 994  T2: 1351 | Uncertain | 279 | 28.1% | 306 | 22.6 |
| Lei et al., 2017 [56] | Shanghai, East | 2013 | Nulliparous women | 1299 | Adhere to doctor’s suggestion | -- | -- | 241 | 18.6 |
|  |  |  | Multiparous women | 260 | Adhere to doctor’s suggestion | -- | -- | 43 | 16.5 |
| Li, 2017 [57] | Jiangsu, East | 2013 | Nulliparous and multiparous women | 140 | Adhere to doctor’s suggestion | -- | -- | 17 | 12.1 |
| Xie, 2017 [63] | Guangdong, East | 2016 | Nulliparous and multiparous women | 244 | Fine with both MOD | -- | -- | 42 | 17.2 |
| Wang, 2017 [60] | Tianjin, East | 2014 | Postpartum women | 582 | Fine with both MOD | 127 | 21.8 | -- | -- |
| Liao, 2017 [64] # | Hunan and Hubei, Central | 2016 | Nulliparous and multiparous women | 400 | Not consider it | N=31 7.8 | | | |

* The preference for a mode of delivery (MOD) was reported during the pregnancy (gestational age not reported)

# This study reported total 31 pregnant women “not consider the mode of delivery yet” without stratification of gestational age.
