# Supplementary material for: Prevalence of and reasons for women’s, family members’, and health professionals’ preferences for cesarean section in China: A mixed-methods systematic review
Source: PLoS Med. 2018 Oct 16;15(10):e1002672. doi: 10.1371/journal.pmed.1002672 (PMC6191094; doi:10.1371/journal.pmed.1002672)
Supplement: S6 Table — (DOCX) [file pmed.1002672.s008.docx]

**S6 Table Reasons for preference for caesarean section reported by women, quantitative studies**

|  | **Preference for current pregnancy or index birth** | | | | | | | | | | | | | **Hypothesized preference** |
| --- | --- | --- | --- | --- | --- | --- | --- | --- | --- | --- | --- | --- | --- | --- |
|  | Cao et al., 2004 [37] | Pang et al., 2007 [8] | Song et al., 2007 [46] | Jiang, 2012 [44] | Zhang et al., 2012* [54] | Li et al., 2014 [36] | Zhang et al., 2016 [50] | Gao et al., 2017 [55] | Huang et al., 2017 [61] | Xu et al., 2017 [62] | Xie et al., 2017 [63] | Wang, 2017 [60] | Zhang et al., 2017 [70] | Loke et al., 2015 [73] |
|  | (Shanghai, East; Parity unknown, n=139) | (Hong Kong, P0, n=52) | (Beijing, East; P0, n=24) | (Zhejiang, East; P0, n=49) | (Hebei, East; Parity unknown, n=57) | (Beijing, East; P0, n=72) | (Jiangxi, Central; parity unknown, n=2913) | (Hunan, Central; parity unknown, n=207) | (Anhui, Central; P0 and P+, n=268) | (Hubei, Central; P0, n=107) | (Guangdong, East; ; P0 and P+, n=37) | (Tianjin, East; P0 and P+, n=105) | (Beijing, East; P0 and P+, n=85) | (Hong Kong, pregnant women or women giving birth within past 3 years, n=73) |
| **Pain-related fear** |  |  |  |  | NR |  |  |  |  |  |  |  |  |  |
| - Fear of labour pain | 42.1% |  | 50.0% | 24.5% |  | 80.6% |  |  | 34.0% | 47.7% |  |  |  | 79.5% |
| - Fear of “twice pain” | 20.0% |  | 70.8% | 48.9% |  |  |  |  | 35.1% | 10.3% |  |  |  |  |
| - Less overall pain |  | 25.0% |  |  |  |  |  |  |  |  | 8.1% | 68.6% | 16.5% |  |
| - Afraid of unable to endure (labour) pain |  |  |  |  |  |  | 27.5% |  |  |  |  |  |  |  |
| - Low pain tolerance |  |  |  |  |  | 15.3% |  |  |  |  |  |  |  |  |
| **Fear of VD -perceived maternal short-term risks** | NR |  |  |  | NR |  | NR |  |  |  |  |  |  |  |
| - Less vaginal trauma |  | 3.8% |  |  |  |  |  |  |  |  |  |  |  |  |
| - Afraid of unable to endure VD |  |  | 33.3% |  |  |  |  | 36.8% |  |  |  |  |  |  |
| - Less hard during childbirth for mother |  |  |  |  |  |  |  |  |  |  | 37.8% | 59.0% |  |  |
| - Good for recovery after birth by CS |  |  | 12.5% |  |  |  |  |  |  |  |  |  |  |  |
| - CS is safe and reliable |  |  |  | 14.3% |  |  |  |  | 16.8% | 25.2% | 45.9% | 55.2% |  |  |
| - Avoidance of emergency CS |  |  |  |  |  |  |  |  |  |  |  |  | 23.5% |  |
| - Fear of perineal cut |  |  |  |  |  | 25.0% |  |  |  |  |  |  |  |  |
| - Safer for mother by CS |  |  |  |  |  | 18.1% |  |  |  |  |  |  | 11.8% |  |
| - Fear of tearing of the perineum |  |  |  |  |  |  |  |  |  |  |  |  | 23.5% | 26.0% |
| - Possible anal/urinary incontinence due to VD |  |  |  |  |  |  |  |  |  |  |  |  |  | 26.0% |
| - To reduce the damage of the pelvic floor |  |  |  |  |  |  |  |  |  |  |  |  | 9.4% |  |
| **Fear of VD - perceived maternal long-term risks** | NR | NR |  |  | NR |  | NR |  |  |  |  |  |  |  |
| - No negative impact on sexual life by CS |  |  | 20.8% | 4.1% |  |  |  |  | 4.9% |  |  |  | 9.4% |  |
| - Better keeping body image by CS |  |  |  |  |  | 1.4% |  |  |  |  |  |  |  |  |
| - Less sexual dissatisfaction by CS |  |  |  |  |  | 1.4% |  |  |  |  |  |  |  | 16.4% |
| - Fear of vaginal relaxation |  |  |  |  |  |  |  |  |  | 9.3% |  |  |  |  |
| **Fear of VD -perceived risks for the baby** |  |  |  | NR |  |  |  |  |  |  |  |  |  |  |
| - Fear of risk for baby | 17.1% |  |  |  |  |  |  | 37.8% |  |  |  |  | 41.2% |  |
| - Ensuring safe of baby |  |  | 45.8% |  | 54.5% |  |  |  |  |  |  |  |  |  |
| - Safer for the baby |  | 44.0% |  |  |  | 18.1% | 7.5% |  |  |  |  |  |  |  |
| - Perceived that baby would be more clever and less cost |  |  | 8.4% |  |  |  |  |  |  |  |  |  |  |  |
| - Healthier baby by CS |  |  |  |  |  | 2.8% |  |  |  |  | 13.5% |  |  |  |
| - Health of the newborn |  |  |  |  |  |  |  |  |  |  |  |  |  | 53.4% |
| - Birth trauma to the newborn |  |  |  |  |  |  |  |  |  |  |  |  |  | 32.9% |
| - Respiratory trauma to the newborn |  |  |  |  |  |  |  |  |  |  |  |  |  | 13.7% |
| **Convenience of planning** | NR |  | NR | NR | NR |  | NR |  |  |  |  |  |  |  |
| - Convenience for sterilisation |  | 3.8% |  |  |  |  |  |  |  |  |  |  |  |  |
| - Better control of the time of birth |  | 5.8% |  |  |  |  |  |  |  |  |  |  |  | 27.4% |
| - Controllability |  |  |  |  |  | 11.1% |  |  |  |  |  |  |  |  |
| - Faster/more convenient method of delivery |  |  |  |  |  |  |  |  |  |  | 29.7% | 64.8% |  | 24.7% |
| - Better planning for paternity leave |  |  |  |  |  |  |  |  |  |  |  |  |  | 15.1% |
| **Cultural and societal related beliefs** | NR | NR |  |  | NR | NR |  |  |  |  |  |  |  |  |
| - Reasonable for schedule and able to select “lucky date” for the birth |  |  |  |  |  |  | 3.5% |  |  |  |  |  |  |  |
| - Choosing an auspicious date |  |  | 12.5% | 8.2% |  |  |  |  | 1.1% | 7.5% |  |  | 10.6% | 19.2% |
| **Medical and other reasons** | NR |  | NR | NR | NR |  |  |  |  |  |  |  |  |  |
| - Having CS medical indications |  |  |  |  |  |  | 50.9% |  |  |  |  |  |  |  |
| - Abnormal detected during prenatal check-up |  |  |  |  |  |  |  | 39.3% |  |  |  |  |  |  |
| - Personal health reasons |  |  |  |  |  | 5.6% |  |  | 29.9% |  |  |  |  |  |
| - Maternal health |  |  |  |  |  |  |  |  |  |  |  |  |  | 45.2% |
| - Newborn's birth presentation |  |  |  |  |  |  |  |  |  |  |  |  |  | 17.9% |
| -Doctors/midwives advice |  | 5.8% |  |  |  | 6.9% |  |  |  |  |  |  |  |  |
| - Women should have the right to choose |  |  |  |  |  |  |  |  |  |  |  |  |  | 19.2% |
| - Medical insurance coverage |  |  |  |  |  |  |  |  |  |  |  |  |  | 2.7% |
| - Large baby |  |  |  |  |  |  |  |  |  |  |  |  |  | 21.9% |
| - Twins/triplets |  |  |  |  |  |  |  |  |  |  |  |  |  | 6.8% |
| - Advanced age for childbirth |  |  |  |  |  |  |  |  |  |  |  |  |  | 30.1% |
| - Quick recovery |  |  |  |  |  |  |  |  |  |  | 5.4% |  |  |  |
| - Prior CS |  |  |  |  |  |  |  |  |  |  |  |  | 17.6% |  |
| - A fashion |  |  |  |  |  |  |  |  |  |  |  |  | 1.2% |  |
| - Prior negative experience from VD |  |  |  |  |  |  |  |  |  |  |  |  | 1.2% |  |

NR: not reported; Fear of “twice pain”: Fear of failure of trial of labour and then having to undergo CS

P0: Nulliparous women; P+: Multiparous women

* The most important reason for the preference for CS was reported during the pregnancy (gestational age was not reported)

^ “Fear of vaginal delivery” (7.7%, 4/52) was one of the reasons for CS preference in this study but there is no detailed explanation on this reason.
