# Supplementary material for: Prevalence of and reasons for women’s, family members’, and health professionals’ preferences for cesarean section in China: A mixed-methods systematic review
Source: PLoS Med. 2018 Oct 16;15(10):e1002672. doi: 10.1371/journal.pmed.1002672 (PMC6191094; doi:10.1371/journal.pmed.1002672)
Supplement: S7 Table — (DOCX) [file pmed.1002672.s009.docx]

**S7 Table Summary of initial concepts, emergent themes, final themes and supporting quotes**

| **Initial concepts** | **Emergent themes** | **Contributing studies** | **Supporting quotes** | **Final themes** |
| --- | --- | --- | --- | --- |
| Perceptions of caesarean section have changed | **Belief caesarean section is now a safe/r option for birth** | 19 studies – [26, 78-95] | “I think nowadays caesarean section is not the same as caesarean section before. After caesarean section previously you were afraid of some complications nowadays technology of caesarean section has improved and also use some new products. I don’t worry about any complications.” (Woman, Guangxi, Jiang et al, 2012)  “An ancient proverb says that “If you pass the crisis [childbirth], you could enjoy stewed chicken with sesame oil. If you failed, you would lie in the coffin.” This showed how hard and dangerous childbirth was.” (Woman, Taiwan, Huang et al 2013b)  “Caesarean section surgery is very successful, without any danger” (Woman, Shanghai, Zhou et al, 2012)  “Out of 15 women who delivered in our village, 14 of them had a caesarean section” (Village FP worker, Anhui, Huang et al 2013a)  “Caesarean section can avoid suffering twice pain” (Woman, Shanghai, Wang et al 2006)  “If you ask me what is my choice of childbirth for next pregnancy, the answer must be caesarean section. It is because it can assure the safety of my baby” (Woman, Hong Kong, Lee 2001).  “Caesarean section is convenient. I don’t need to push and it’s quick. I handover everything to my doctor” (Healthcare provider and mother, Shanghai, Wang and Ding, 2013)  My husband worried after my vaginal delivery my body image would change. He wished me to keep my body sexually attractive” (Woman, Guangxi, Jiang 2012)  “We didn’t consider to give birth by myself since the beginning because the date of pregnancy was selected so the date of birth was also selected. My family believe our birth time, day and year are lucky.” (Woman, Shanghai, Zhou et al, 2012)  “I selected to have my surgery in the morning at 9 o’clock on 6th of February.” (Women, Shanghai, Chen et al 2008)  “So I would rather choose an auspicious day beforehand for caesarean delivery.” (Woman, Gu, 2018) | **Beliefs about caesarean section** |
| Belief no longer necessary to labour and have emergency caesarean (twice pain) |  |  |  |  |
| Positive views of caesarean section compared to vaginal birth | **Belief caesarean section has social and cultural advantages for birth** | 13 studies – [26, 78-80, 82, 84-87, 89, 92- 94] |  |  |
| Negative views of labour and vaginal birth in contrast to caesarean section |  |  |  |  |
| Social and cultural convenience of birthing to time (work scheduling, school year, auspicious dates) |  |  |  |  |
| Women’s preferences informed by availability (i.e. what they or insurance can pay) | **Financial drivers, financial means and financial burdens and caesarean section** | 10 studies – [26, 81, 82, 84-88, 91, 93] | “Profit from caesarean section surgery is much higher than vaginal delivery. From the perspective of the hospital we want to reduce caesarean section rate but we cannot do anything” (Township Health Centre Manager, Anhui, Liu et al 2010)  ““I think that one aim of caesarean section is to decrease doctors’ trouble, and the other is to get more profit for the hospital.” (Village FP worker, Anhui, Huang et al 2013a)  “I think current economic situation means can afford expenditure of caesarean section” (Woman, Shanghai, Zhou et al, 2012)  “It’s like the family economic situation is not really associated with a choice of type of birth. In my district the overall economic situation is good and there are some good encouragement policies. Rural pregnant women can afford caesarean section” (Township Health Centre Manager, Anhui, Liu et al 2010)  “ELCD is a big burden for us. We need to pay full expenses by ourselves, including the operation fee, anaesthetic fee, and others” (Woman, Taiwan, Huang et al 2013b).  “We have to do [caesarean section] because pregnant women and their family think caesarean section can guarantee safety of both mother and baby. If there are any adverse birth outcomes the hospital can avoid responsibility” (Township Health Centre Manager, Anhui, Liu et al 2010)  “Nowadays, there were many phenomena of “Yi Nao” [healthcare disturbance were families attack doctors]). The hospitals suffered from loss. Pregnant women often made decision by themselves on when they would like to have CS. If doctors suggested not having CS, pregnant women and their family members put pressures on doctors. Performing CS was a self-protection approach for doctors. (Healthcare Manager, Hunan, Zhu et al 2013)  “I feel caesarean section is safer than vaginal delivery because everything is visible” (Woman , Liuzhou, Chen et al 2008) | **Healthcare system factors** |
| Financial income more important than health outcomes |  |  |  |  |
| Yi Nao (healthcare disturbance) and medico-legal concerns | **Mistrust between women and healthcare professionals** | 8 studies – [79-84, 88, 92] |  |  |
| Mistrust in hospitals, Drs and MWs |  |  |  |  |
| Lack of Drs and MWs skills during labour and vaginal birth | **Quality of care: Health professionals’ training, skills, experience and influence** | 13 studies – [81-84, 86-88, 90-95] | “Obstetricians are familiar with the operation. Combined with a shortage of skilled midwives, and the doctors’ poor skills to attend vaginal delivery and manage dystocia. CS may not cause more morbidity or mortality for women and babies than a normal delivery.” (Village FP worker, Anhui, Huang et al 2013a)  “The charge for CS was high. Under the profit driving, CS rate increased. However, fee for natural delivery was only 100 yuan, which could not reflect value of midwives” (Healthcare Manager, Hunan, Zhu et al 2013)  Skills for birth attendants [in vaginal delivery] have decreased. With the increase of caesarean section rate young doctors in their routine work and in-service training in higher level hospitals have attended less vaginal deliveries. However, technology of caesarean section has over time has advanced but the skills of birth attendants in vaginal delivery have decreased. Young doctors would like to perform caesarean section so high caesarean section rate falls into a circle” (Township Health Centre Manager, Anhui, Liu et al 2010)  “Midwives had few training opportunity, and professional skills could not be improved. Management of labour ward was poor“(Healthcare Manager, Hunan, Zhu et al 2013).  “My mother and my husband were there from the labour until I gave birth to the baby. I could bear the pain when my husband and mother were with me, otherwise I cannot.” (Woman, Anhui, Raven et al 2015).  “Nobody cared about you when you suffered from pain over ten hours. If you felt very pain and shout, the doctor would complain. It was impossible to have relatives companion. Psychologically, it was too cruel, especially for pregnant women.” (Woman, Beijing, Ji 2006)  “The skills of labour support are not well developed. Rate of successful painless delivery is not high leading to caesarean section in the end. If there will be a safe and effective method of pain relief many pregnant women would like to choose vaginal delivery.” (Township Health Centre Manager, Anhui, Liu et al 2010)  “I hope that when I feel pain I can have some pain relief, but this is not possible. Otherwise there would not be so many women choosing caesarean section.” (Woman, Anhui, Raven et al 2015) |  |
| Too little value placed on Midwifery care (skills, training, profession, financial reward) |  |  |  |  |
| Preference for caesarean section to avoid lack of pain relief and support during labour and vaginal birth (includes episiotomy) | **Quality of care: Availability of labour support and pain relief during vaginal birth** | 11 studies – [26, 79, 81-83, 86-89, 91, 93] |  |  |
| Confidence in hospitals, Drs and MWs provision of labour care |  |  |  |  |
| Women’s reproductive right to choose reform | **Complexity and autonomy: Women’s right to choose caesarean section in China** | 16 studies – [26, 79-89, 91-94] | “I have the right to make choices for myself.” (Woman, Hong Kong, Lee et al 2001)  “I wished to give birth by myself but I was afraid I couldn’t make it, and there might be complications then in the end I might have to undergo caesarean section. The risks of suffering twice I decided to choose caesarean section directly.” (Woman, Guangxi, Jiang et al 2012)  “Informed choice means you are allowed to choose caesarean section.” (Woman, Shanghai, Chen et al 2008)  “I want to choose caesarean section. The doctor thought there was no medical indication so refused. I argued with her and then I reminded her I have a right to choose.” (Woman, Shanghai, Chen et al 2008)  “Initially, I purposively proposed the operation to be done when I went into labour because it meant the baby was mature enough to be born. However, my mother-in-law strongly suggested I to choose a ‘lucky day for the baby’s birthday” (Woman, Hong Kong, Lee et al 2001)  “In China the regulation of protection of maternal and child right says every woman has the right to choose her own mode of delivery. Undoubtedly this law is helpful to protect Chinese woman to seek for maternal health services, however the promotion and explanation of the law is facing many challenges” (Healthcare Provider 3, Liuzhou, Chen et al 2008).  “Of course there is still someone who told me that this decision was not appropriate and caesarean delivery still carried great risks, but I am not worried about that. Caesarean is easy surgery for obstetricians, they would assist me to accomplish it, and doctors are intelligent people, they can handle it.” (Woman, Taiwan, Huang et al 2013b)  “The caesarean rate was higher before because of social factors. Women would get a caesarean just because... now all these will try for vaginal birth.” (Nurse-midwife, Wang and Hesketh 2017)  “I worried about the safety of the baby so I choose caesarean section. Anyway I only give birth once in my life” (Woman, Shanghai, Zhou et al, 2012)  “I have been waiting for this moment for 5 years already. Therefore, I must do everything that can ensure the safety of my daughter because I may not be pregnant again. I cannot afford any risk.” (Woman, Hong Kong, Lee et al 2001)  “I don’t want to take a risk. Anyway I only give one birth. So I choose caesarean section.” (Woman, Guangxi, Jiang et al 2012)  “Since my childhood my parents loved me very much. I ‘m a relatively gentle person. I think I couldn’t bear vaginal delivery (Woman, Shanghai, Zhou et al, 2012)  “I thought I would only have one child!” “At the time we were not allowed a second child so we never considered the risks.” (Multiparous woman (requested CS 9 years ago) and her husband, Wang and Hesketh 2017)  “Now we are so lucky to have the new two-child policy… That’s why I had planned a trial of labour for the first child.” (Woman, Gu et al, 2018) | **Societal context and social change** |
| Choice and socio-economic status |  |  |  |  |
| Choice and Dr-patient relationship |  |  |  |  |
| Choice and change in preference |  |  |  |  |
| Choice and responsibility for health |  |  |  |  |
| Deep rooted fear of labour pain and natural childbirth |  |  |  |  |
| Safety of only baby paramount | **Safety of baby paramount concern of women and families** | 15 studies – [26, 78, 79, 81-85, 87-89, 92-95] |  |  |
| All women are different (physical stature, experiences, emotions) | **Women's experiences: How women birth is a fundamental human concern of individuals and society** | 15 studies – [26, 78, 79, 81-83, 85, 87-90, 92-95] | “I was impacted by others, for example, friends, colleagues. They had CS and it was safe. Everyone thought it was fine. You could choose time and had no pain” (Woman, Beijing, Ji, 2006)  “I want a caesarean delivery because Dee Hsu [a well-known female star in Taiwan] said that childbirth affects the tightness of vagina “(Woman, Taiwan, Huang et al 2013b)  “They’ve [the hospital] done health promotion pretty well. They really push natural birth. They constantly recommend natural birth, especially for the first child.” (Woman, Wang and Hesketh, 2017)  “Many pregnant women come from Yunnan, Guizhou, Sichuan [Western, poorest, least developed provinces] and give birth in our hospital. All of them choose vaginal delivery. They have a strong survival capacity. It may be associated with poor living conditions however local pregnant women had over-acted when they had the trial of labour and couldn’t bear the pain” (Township Health Centre Manager, Anhui, Liu et al 2010)  “During my pregnancy my weight increased 15KG. I looked at some information. Overweight pregnant women might have complications such as hypertension, diabetes, and other cardiovascular disease so I choose caesarean section it might be safer.” (Woman, Jiang et al 2012)  “I heard that NSD is painful before birth, but that caesarean delivery is painful afterwards: I worry that the pain after delivery would disturb me so that I cannot take care of my baby, like breastfeeding or interacting with him” (Woman, Hong Kong, Lee et al 2001).  “Before my pregnancy, I had a fibroid so that’s why I would like to be pregnant and then I thought I would have surgery for a birth and then together remove it” [the baby and the fibroid]. (Healthcare provider and mother, Wang & Ding, 2013)  “Caesarean section can avoid suffering twice pain and baby’s head will not be crushed is an advantage for baby. However, it’s not good for baby’s breathing” (Woman, Shanghai, Wang et al 2006)  “I planned it to give birth by myself. I thought it would be better for baby. But I was admitted into the hospital for birth. My blood pressure was high, probably because of nervousness or maybe other reason. I worried so I chose caesarean section” (Woman, Shanghai, Zhou et al, 2012)  “After caesarean section we found the cord didn’t wrap around the baby’s neck. I thought baby was too young to come out. I wanted to choose a safe mode of delivery for baby. In the end he suffered more. I was regretful” (Healthcare provider and mother, Shanghai, Wang & Ding, 2013)  “I wonder why my sister-in-law did not feel any pain after a caesarean operation. I think it may be due to individual difference. The wound is quite unendurable” (Woman, Hong Kong, Lee et al 2001).  “Some people said it will be very exciting to see your baby being born. It seems to me that I have missed something but I cannot tell you what it is” (woman, Hong Kong, Lee 2001). | **Women’s experiences** |
| Influence of information about birth from friends, family, colleagues, net pals, books, celebrities |  |  |  |  |
| Contradictions and commonalities in other women’s birth stories | **Women's experiences: Heterogeneity, uncertainty, and unresolved meaning surrounding birth** | 18 – studies [26, 78-85, 87-95] |  |  |
| Women’s negative views of caesarean section |  |  |  |  |
| Positive and negative views of vaginal birth |  |  |  |  |
| Doubt which birth mode is best |  |  |  |  |
