# Supplementary material for: Prevalence of and reasons for women’s, family members’, and health professionals’ preferences for cesarean section in China: A mixed-methods systematic review
Source: PLoS Med. 2018 Oct 16;15(10):e1002672. doi: 10.1371/journal.pmed.1002672 (PMC6191094; doi:10.1371/journal.pmed.1002672)
Supplement: S1 Text — (PDF) [file pmed.1002672.s010.pdf]

PROSPERO International prospective register of systematic reviews

Review title and timescale

1 Review title

Give the working title of the review. This must be in English. Ideally it should state succinctly the interventions or exposures being reviewed and the associated health or social problem being addressed in the review.

Women's and healthcare providers' preference for caesarean section: a mixed methods systematic review

2 Original language title

For reviews in languages other than English, this field should be used to enter the title in the language of the review. This will be displayed together with the English language title.

3 Anticipated or actual start date

Give the date when the systematic review commenced, or is expected to commence.

14/03/2016

4 Anticipated completion date

Give the date by which the review is expected to be completed.

31/03/2017

5 Stage of review at time of this submission

Indicate the stage of progress of the review by ticking the relevant boxes. Reviews that have progressed beyond the point of completing data extraction at the time of initial registration are not eligible for inclusion in PROSPERO. This field should be updated when any amendments are made to a published record.

The review has not yet started

Review stage	Started	Completed
Preliminary searches	Yes	No
Piloting of the study selection process	Yes	No
Formal screening of search results against eligibility criteria	No	No
Data extraction	No	No
Risk of bias (quality) assessment	No	No
Data analysis	No	No

Provide any other relevant information about the stage of the review here.

Review team details

6 Named contact

The named contact acts as the guarantor for the accuracy of the information presented in the register record.

Qian Long

7 Named contact email

Enter the electronic mail address of the named contact.

longq@who.int

8 Named contact address

Enter the full postal address for the named contact.

20 Avenue Appia 1211 Geneva 27, Switzerland

9 Named contact phone number

Enter the telephone number for the named contact, including international dialing code.

+41227913470

10 Organisational affiliation of the review

Full title of the organisational affiliations for this review, and website address if available. This field may be completed as 'None' if the review is not affiliated to any organisation.

World Health Organization

Website address:

11 Review team members and their organisational affiliations

Give the title, first name and last name of all members of the team working directly on the review. Give the organisational affiliations of each member of the review team.

Title	First name	Last name	Affiliation
Dr	Qian	Long	Department of Reproductive Health and Research, World Health Organization
Dr	Ana Pilar	Betran	Department of Reproductive Health and Research, World Health Organization
Dr	Meghan	Bohren	Department of Reproductive Health and Research, World Health Organization
Professor	Maria Regina	Torloni	Department of Obstetrics, Federal University of Sao Paulo

12 Funding sources/sponsors

Give details of the individuals, organizations, groups or other legal entities who take responsibility for initiating, managing, sponsoring and/or financing the review. Any unique identification numbers assigned to the review by the individuals or bodies listed should be included.

The reviewers have no support or funding to report. The reviewers will be personally salaried by their respective institutions

during the period of conducting and writing the results of the review, though no specific salary was set aside or given for the conduct of this review.

13 **Conflicts of interest**

List any conditions that could lead to actual or perceived undue influence on judgements concerning the main topic investigated in the review.

Are there any actual or potential conflicts of interest?

None known

14 **Collaborators**

Give the name, affiliation and role of any individuals or organisations who are working on the review but who are not listed as review team members.

Title	First name	Last name	Organisation details

Review methods

15 **Review question(s)**

State the question(s) to be addressed / review objectives. Please complete a separate box for each question.

a) To identify, appraise and synthesize qualitative and quantitative research evidence on women's, healthcare providers' and administrator's, and other key stakeholders' perceptions and preferences regarding CS as a mode of delivery;
b) To identify and map factors motivating preferences for CS, including societal, cultural, financial and individual factors;
c) To explore how the findings of this review can enhance our understanding of underlying factors driving the worldwide increase of CS.

16 **Searches**

Give details of the sources to be searched, and any restrictions (e.g. language or publication period). The full search strategy is not required, but may be supplied as a link or attachment.

The following databases will be searched: PubMed, EMBASE, Social Science Index citations, CINAHL, Global health library, LILACS, CNKI.

We will search the web (http://www.opengrey.eu/) to identify relevant grey literature.

In addition to electronic search strategy, we will screen the references of all included studies and contact experts in relevant fields of study in order to capture the largest number of publications on this topic.

There will be no language or geographic restrictions for the search. We will include studies published from January 1, 1990 to present.

17 **URL to search strategy**

If you have one, give the link to your search strategy here. Alternatively you can e-mail this to PROSPERO and we will store and link to it.

I give permission for this file to be made publicly available

Yes

18 **Condition or domain being studied**

Give a short description of the disease, condition or healthcare domain being studied. This could include health and wellbeing outcomes.

In the context of the unprecedented rise in caesarean section rates seen worldwide over the last few decades, we study the preferences and opinions of women, healthcare providers and managers and policy makers on caesarean section as a mode of delivery.

19 **Participants/population**

Give summary criteria for the participants or populations being studied by the review. The preferred format includes details of both inclusion and exclusion criteria.

Studies reporting preferences and opinions of both health users and health supply side will be eligible for inclusion:

• Women regardless of their obstetric characteristics (e.g. parity, their pregnancy status and whether or not they have had a previous CS), and regardless their marital or socio-economic status. Results will be reported stratified and clearly differentiated when possible.

• Health managers and healthcare providers who are working in maternity services within public and private sectors or mixed public and private delivery system.

• Policy-makers, or other relevant individuals or groups involved in developing or implementing policies, interventions or organisation of childbirth care (e.g. programme managers, administrative staffs etc.).

20 **Intervention(s), exposure(s)**

Give full and clear descriptions of the nature of the interventions or the exposures to be reviewed

Intervention/exposure is not applicable for this systematic review. The phenomenon of interest is preference and opinions of women, policy makers, health managers and healthcare providers on caesarean section as a mode of delivery.

21 **Comparator(s)/control**

Where relevant, give details of the alternatives against which the main subject/topic of the review will be compared (e.g. another intervention or a non-exposed control group).

Comparators/control is not applicable for this systematic review.

22 **Types of study to be included initially**

Give details of the study designs to be included in the review. If there are no restrictions on the types of study design eligible for inclusion, this should be stated.
This review will include original research which investigated women preferred mode of delivery or providers' preferences and the reasons for such preferences. Studies conducted in both urban and rural settings without any restrictions on the country's level of development (including low-, middle- and high-income countries) will be eligible.
• Individual surveys reporting reasons of preference for CS
• Qualitative studies that: a) use recognised methods (in-depth interviews, focus group discussions, or observation); and b) describe the methods used in analysis including, but not limited to framework analysis, thematic analysis, content analysis, grounded theory and constant comparison)
• Mixed quantitative and qualitative studies whose quantitative and qualitative methods meet the criteria mentioned above.

Criteria for exclusion include:
• Studies based on health facility register data or second hand data analysis.
• Studies that use qualitative methods for data collection but which do not use appropriate methods to perform the analysis.
• Studies where there was no information on the study design, study population and how data were collected.

23 Context

Give summary details of the setting and other relevant characteristics which help define the inclusion or exclusion criteria.

24 Primary outcome(s)

Give the most important outcomes.
The main outcomes of interest are proportions of participants (women, healthcare providers and managers and policy makers) preferred for a CS as a mode of delivery and the reasons for those preferences.
Give information on timing and effect measures, as appropriate.

25 Secondary outcomes

List any additional outcomes that will be addressed. If there are no secondary outcomes enter None.
None

Give information on timing and effect measures, as appropriate.

26 Data extraction, (selection and coding)

Give the procedure for selecting studies for the review and extracting data, including the number of researchers involved and how discrepancies will be resolved. List the data to be extracted.
All citations identified from the electronic searchers will be downloaded into EndNote and duplicates deleted. Two reviewers will independently screen the title and the abstract of all identify citations and select potentially relevant citations for full-text review. Two reviewers will independently and in duplicated read and assess each full text article and those fulfilling the aforementioned criteria will be included in the review. Any disagreement and uncertainties will be resolved by discussion, the involvement of a third reviewer, or both.

A data extraction form will be developed and used to extract the following information: • Study setting (city, country, urban or rural, community or type and level of health facility) and study year • Study participants (e.g. demographic and socio-economic characteristics of women, demographic and professional characteristics of healthcare providers) and sample size • Type of study/study design • Description of methods of data collection and data sources • Data analysis • Outcomes of interest: preferred mode of delivery and reasons for preference • Contextual issues (which could explain the preferences for a mode of delivery by study authors, for example social norms, health policy related to obstetric care, health financing and health services organization and delivery) • Author conclusions and/or recommendations

27 Risk of bias (quality) assessment

State whether and how risk of bias will be assessed, how the quality of individual studies will be assessed, and whether and how this will influence the planned synthesis.
Quality of included studies will be assessed by two reviewers independently:
• The quality of quantitative studies will be assessed according to the "Strengthening the Reporting of Observational Studies in Epidemiology" (STROBE) statement. The assessment domains will include: eligibility criteria, method assessment, participant characteristics, outcome measures, discussion of sources of bias.
• The quality of qualitative studies will be assessed using an adaptation of the Critical Appraisal Skills Programme (CASP) quality-assessment tool (http://www.casp-uk.net).

28 Strategy for data synthesis

Give the planned general approach to be used, for example whether the data to be used will be aggregate or at the level of individual participants, and whether a quantitative or narrative (descriptive) synthesis is planned. Where appropriate a brief outline of analytic approach should be given.
Both quantitative and qualitative data will be synthesized:

a) Quantitative data
Due to the anticipated high heterogeneity between study designs and population, it is unlikely that we will be able to perform a meta-analysis. The percentages of reported reasons of women preference for CS will be sorted by identified themes in correspondence with qualitative findings (see below) and presented as a narrative brief.

b) Qualitative data
A thematic synthesis approach will be used to analyse and synthesize the qualitative data. Thematic synthesis is comprised of a three step process: Stage 1 & 2 – coding and development of descriptive themes: A spreadsheet will be created to extract qualitative data from the primary findings. Codes will first be structured as "free" codes with no established link between them. As each study is coded, the reviewers will be able to translate concepts from one study to another. This will further develop the codebook, and new codes will be added as necessary. Reviewers will seek similarities and differences between the codes and group the codes according to a hierarchical structure. In the first two

stages, two reviewers will independently code the findings, the work as a team to generate analytical themes in stage 3. Stage 3 – generating analytical themes: In this stage, the reviewers will conduct analytical discussions on these themes to generate interpreted themes. This is a cyclical process and will be repeated until the themes generated are sufficiently conceptual to explain and describe the initial descriptive themes from stage 2.

In addition, we will use the CERQual (Confidence in the Evidence from Reviews of Qualitative research) approach to assess the confidence we have in the findings from included studies. This approach, building on the GRADE tool for Cochrane effectiveness reviews is a work in progress, but is becoming the standard to assess the confidence of the findings from qualitative evidence syntheses.

The CERQual approach assess four concepts:
a) Methodological limitations of included studies: This refers to the extent in which there are weaknesses in the design or methodology of studies that contributed evidence to a review finding. Confidence in a finding may be lowered by major methodological limitations.
b) Relevance of the included studies to the review question: This refers to the extent to which the primary studies supporting a review findings are applicable to the context (setting, participants, phenomenon of interest) specified in the review question. Confidence in a finding may be lowered when the listed contextual issues of a primary study used to support a review finding are different to the context of the review question.
c) Coherence of the review finding: This points to the extent in which there are patterns identified across the review findings contributed by each included study. This can be where a review finding is consistent across more than one context, or a finding that includes an explanation/s for variation/s across studies. Variations in data across the included studies without convincing explanations may lower the confidence of a review finding.
d) Adequacy of the data contributing to a review finding: This refers to an assessment of the level of richness, scope and quantity of data, which support a review finding. Confidence in a finding may be lowered if a finding is supported by results from only one or a few of the included studies, or when the data supporting a finding are very thin.

The above assessments will result in an assessment of the overall confidence of each review finding as high, moderate, low or very low. Qualitative review findings and CERQual assessments will be presented in a Summary of Qualitative Findings Table and Evidence Profile that summarizes the finding, overall confidence assessment, and rationale for assessment of each finding.

The differences between the review authors regarding subject expertise, employment and other background factors may affect the manner in which we collect, analyse and interpret the data. We will account for these differences and any other issues that may contribute to the interpretation of the review findings, by describing it in a “reflexivity” section when publishing the review results.

29 Analysis of subgroups or subsets

Give any planned exploration of subgroups or subsets within the review. ‘None planned’ is a valid response if no subgroup analyses are planned.

Subject to identified studies, subgroup analysis by the country's level of development (including low-, middle- and high-income countries) will be conducted.

Review general information

30 Type of review

Select the type of review from the drop down list.

Epidemiologic

31 Language

Select the language(s) in which the review is being written and will be made available, from the drop down list. Use the control key to select more than one language.

English

Will a summary/abstract be made available in English?

Yes

32 Country

Select the country in which the review is being carried out from the drop down list. For multi-national collaborations select all the countries involved. Use the control key to select more than one country.

Switzerland

33 Other registration details

Give the name of any organisation where the systematic review title or protocol is registered together with any unique identification number assigned. If extracted data will be stored and made available through a repository such as the Systematic Review Data Repository (SRDR), details and a link should be included here.

34 Reference and/or URL for published protocol

Give the citation for the published protocol, if there is one.

Give the link to the published protocol, if there is one. This may be to an external site or to a protocol deposited with CRD in pdf format.

I give permission for this file to be made publicly available

Yes

35 Dissemination plans

Give brief details of plans for communicating essential messages from the review to the appropriate audiences.

Do you intend to publish the review on completion?
Yes

36 Keywords
Give words or phrases that best describe the review. (One word per box, create a new box for each term)
Preferences Caesarean section
Mode of delivery
Health users
Health supply
Mixed methods

37 Details of any existing review of the same topic by the same authors
Give details of earlier versions of the systematic review if an update of an existing review is being registered, including full bibliographic reference if possible.

38 Current review status
Review status should be updated when the review is completed and when it is published.
Ongoing

39 Any additional information
Provide any further information the review team consider relevant to the registration of the review.

40 Details of final report/publication(s)
This field should be left empty until details of the completed review are available.
Give the full citation for the final report or publication of the systematic review.
Give the URL where available.

Proposed dummy tables for quantitative data analysis

Table 1 Summary and main characteristics of the included studies

	Quantitative studies	Qualitative studies	Total
**Language of the publication**			
English			
Chinese			
**Region**			
Mainland China ^			
- East			
- Central			
- West			
- Not specified			
HongKong and Taiwan			
**Setting**			
Urban and peri-urban			
Urban and Rural			
Rural			
Not specified			
**Year of data collection**			
2000-2005			
2006-2010			
2011-2016			
Unknown			
**Participants**			
Childbearing women			
- Nulliparous			
- Nulliparous and multiparous			
- Not specified			
Health professionals			
**Quality assessment**			
Low			
Middle			
High			

**Preference for a mode of delivery**			
Preference for index birth			
- During pregnancy			
- Postpartum			
General preference among women of childbearing age			
Maternity care providers' preference for their own birth			
Women reported reasons for preference of a MOD			

^ The mainland China is grouped into East (and East North), Central and West regions according to socio-economic development status (http://www.stats.gov.cn/ztjc/zthd/sjtjr/dejtjkfr/tjkp/201106/t20110613_71947.htm)

Table 2 Women reported preference for CS for index birth during pregnancy and after birth, quantitative studies

Studies	Region	Data collection	Participants	Number of participants	T1: Early or middle pregnancy		T2: Late pregnancy		T3: Postpartum	
					n	%	n	%	n	%
**Longitudinal studies**										
**Cross-sectional studies**										

Table 3 Women reported preference for vaginal delivery for index birth during pregnancy and after birth, quantitative studies

Studies	Region	Data Collection	Participants	Number of participants	T1: Early or middle pregnancy		T2: Late pregnancy		T3: Postpartum	
					n	%	n	%	n	%
**Longitudinal studies**										
**Cross-sectional studies**										

Table 4 Women reported having "no preference" for index birth during pregnancy and after birth, quantitative studies

Studies	Region	Data collection	Participants	Number of participants	"No preference"	Early or middle pregnancy		Late pregnancy		Postpartum	
						n	%	n	%	n	%
**Longitudinal studies**											
**Cross-sectional studies**											

Table 5 Women reported reasons for preferring CS, quantitative studies

Reasons	Preference for index birth									General preference
	Early or middle pregnancy			Late pregnancy				Postpartum		
**Studies**										
Fear of labor pain										
Fear of VD										
Fear of “twice pain”										
Perceived safe and other benefits for baby										
Perceived safe and other benefits for mother										
Less negative impact on sexual life										
Choosing an auspicious date										
Better planning for a birth										
Doctors/midwives advice										
Potential health reasons										
Others										

Table 6 Women reported reasons for preferring VD, quantitative studies

**Reasons**	**Preference for index birth**									**General preference**
	**Early or middle pregnancy**			**Late pregnancy**				**Postpartum**		
**Studies**										
Quick recovery										
Benefits for both mother and baby										
Perceived safe and other benefits for baby										
Perceived safe and other benefits for mother										
Less expenditure										
Natural way to delivery										
Others										
